# Strand-Specific RNA-Seq Analysis of the *Chryseobacterium* sp. HGX-24 Transcriptome in Response to Cadmium Stress

**DOI:** 10.3390/microorganisms14050957

**Published:** 2026-04-23

**Authors:** Qiyu Gao, Zixia Xu, Lin Xu, Wanting Wang, Na Wang

**Affiliations:** 1 Synthetic Biology Engineering Laboratory of Henan Province, School of Life Science and Technology, Henan Medical University, Xinxiang 453003, China; xuzixia2021@163.com (Z.X.); 15737381053@163.com (L.X.); 17639308362@163.com (W.W.); 18530703081@163.com (N.W.); 2 Gansu Key Laboratory of Biomonitoring and Bioremediation for Environmental Pollution, Institute of Microbiology, School of Life Sciences, Lanzhou University, Tianshui Road No. 222, Lanzhou 730000, China

**Keywords:** cadmium contamination, cadmium resistance mechanisms, cadmium resistance genes, plant-microbe synergistic remediation

## Abstract

With the rapid progression of global industrialization and urbanization, heavy metal contamination has emerged as a major global threat, especially cadmium pollution. Consequently, optimizing remediation measures has become a pivotal means to solve cadmium contamination. Compared to traditional physical and chemical remediation methods, microbial remediation has great potential in addressing cadmium pollution. In this study, a novel bacterial strain, *Chryseobacterium* sp. HGX-24, exhibiting high cadmium resistance was successfully isolated and screened from cadmium-contaminated environments. A preliminary discussion of the response mechanisms of this strain under cadmium stress is provided. Additionally, preliminarily explored the synergistic remediation of microbial-plant in cadmium-contaminated soil. Under conditions of high cadmium concentration, cadmium ions were effectively adsorbed by strain HGX-24 through extracellular polymers and functional groups on the cell wall surface, including −COOH, −CONH−, −NH, −OH, and >C=O. Extracellular proteins and polysaccharides were secreted by strain HGX-24 to regulate the adverse effects of heavy-metal cadmium ions on bacterial growth. Furthermore, the expression of genes such as antioxidant defense and ROS scavenging (katG, fabG, ybjT), Fe-S cluster assembly (sufB, sufD), sulfur metabolism (cysAU), amino acid metabolism (hisA, cysD, aspC), phenylacetic acid catabolism (paaC), and ribosomal proteins (rplC, rpsC, rpsL, rplA, rplY, rpmC) was regulated, affecting the synthesis and metabolism of membrane transporters (ABC transporters and efflux RND transporters), antioxidant enzymes (SOD, COT, POD), Fe-S clusters, thioredoxin family proteins, and ribosomal proteins, thereby enhancing resistance to cadmium toxicity. Moreover, strain HGX-24 was found to regulate the activities of redox enzymes in *Zea mays* L., thereby alleviating oxidative stress and reducing the negative feedback effects of reactive oxygen species in *Z. mays*.

## 1. Introduction

In recent years, cadmium has entered the environment through both natural processes—such as sandstorms, sea salt aerosols, volcanic activity, and wildfires—and anthropogenic activities, including metal ore mining, weathering, erosion, smelting, chemical manufacturing, pesticide production, and improper discharge of industrial wastewater [1,2,3,4]. These inputs have caused widespread contamination of soil, water, atmosphere, and the food chain, posing a significant threat to human health and ecosystems [5]. According to recent surveys [6,7], approximately 278,600 hectares of cultivated land in China are contaminated by heavy metals, including cadmium, arsenic, chromium, and lead, accounting for roughly 20% of the total arable area. Cadmium pollution is predominantly concentrated in central and southern provinces, such as Hunan, Guangdong, Yunnan, and Guangxi [8]. In industrially developed regions, soil cadmium contamination often exhibits a watershed-scale spatial pattern.

Cadmium pollution poses a severe threat to human health and ecosystems. In humans, cadmium has a long biological half-life, typically ranging from 10 to 30 years [9], which leads to its progressive accumulation and chronic toxicity. This can cause significant damage to multiple organs, including the kidneys, bones, and respiratory system, and is associated with an increased risk of cancer and osteoporosis [10,11]. At the cellular level, Cd^2+^ disrupts calcium signaling pathways and markedly inhibits antioxidant systems such as glutathione (GSH), superoxide dismutase (SOD), peroxidase (POD), and catalase (CAT) [12], resulting in oxidative stress, DNA damage, and cellular dysfunction. These toxic effects highlight the urgent need for effective remediation strategies for cadmium-contaminated environments.

Compared with traditional physical [13] and chemical remediation [14], microbial-based bioremediation [15] is considered to offer significant advantages in terms of environmental friendliness, cost-effectiveness, and operational simplicity for the remediation of cadmium-contaminated soil and wastewater, although complete removal of cadmium is often challenging. In addition, phytoremediation using plants has also been shown to be effective for cadmium uptake and accumulation in both soil and aquatic environments [16,17]. At present, various microorganisms capable of remediating cadmium contamination have been identified, including bacteria, fungi and actinomycetes, among which bacteria are the most prevalent. These bacteria mainly immobilize Cd^2+^ through biosorption, intracellular sequestration, and biomineralization, thereby reducing its mobility and bioavailability in the environment [18].

Transcriptomics serves as the foundation for functional annotation and mining of all genes expressed in cells under specific environmental conditions [19]. Differential gene expression analysis employs statistical methods to identify genes with significant expression changes between experimental groups, thereby revealing alterations associated with various physiological or pathological states through evaluation of expression fold changes and statistical significance. This approach enables an in-depth understanding of the underlying regulatory mechanisms in biological systems. Microbial cells can effectively immobilize cadmium ions (Cd^2+^) in soil and water through mechanisms such as extracellular adsorption [20], intracellular precipitation [21], mineralization precipitation [22], lysis activation [23], and binding fixation [24]. For instance, *Bacillus subtilis* has been shown to immobilize cadmium via lipid and amino acid metabolism pathways [25]. Similarly, the phosphate-mineralizing strain PMB-5 utilizes phosphorus metabolism genes (e.g., *pst*, *phn*, *ugp*, *ppk*) and heavy metal tolerance genes (e.g., *czcD*, *zntA*, *mgtA*, *katE*, *SOD2*, *dsbA*, *cysM*) during biomineralization to induce the formation of cadmium-amorphous phosphate precipitates [26].

Through the determination of the growth curves of the HGX-24 strain under different cadmium concentrations, it was proved that this strain has high cadmium resistance. However, the underlying cadmium resistance and stress response mechanisms remain poorly understood. Therefore, in this study, the complete transcriptome of strain HGX-24 under cadmium stress was sequenced and annotated. Differentially expressed genes and associated metabolic pathways were systematically identified and analyzed to elucidate the molecular basis of cadmium tolerance. To further evaluate the practical application potential of this strain, pot experiments were conducted using *Zea mays* L. to investigate the effects of HGX-24 inoculation on *Z. mays* growth and cadmium accumulation under different soil Cd concentrations. These experiments aimed to verify whether the cadmium-resistant mechanisms identified at the transcriptomic level could effectively alleviate Cd toxicity and promote the growth of *Z. mays* in a real soil–plant system. These findings provide a theoretical foundation for further optimization and engineering of the cadmium resistance capacity of strain HGX-24, as well as its potential application in the bioremediation of cadmium-contaminated soil for *Z. mays* cultivation.

## 2. Materials and Methods

### 2.1. Experimental Strain and Growth Performance Under Cadmium Stress

The HGX-24 strain (CCTCC No. PB 2026003), isolated and maintained in our laboratory, served as the experimental strain. A single colony was inoculated into 100 mL of sterilized Luria–Bertani (LB) broth (containing 5 g/L yeast extract, 10 g/L NaCl, and 10 g/L tryptone; pH 7.0–7.3) and incubated with shaking (180 rpm, 30 °C) for 16 h to prepare the seed culture, which reached a concentration of (6.5 ± 1.2) × 10^9^ CFU/mL.

To investigate the growth performance of strain HGX-24 under different cadmium concentrations, six bottles of LB medium (50 mL per bottle) were prepared and autoclaved at 121 °C for 20 min. Cadmium (Cd) concentration gradients of 0, 50, 150, 250, 350, and 450 mg/L were then established. Groups A, B, and C served as triplicate parallel treatments. In addition, three bottles of LB medium without bacterial inoculation and without Cd were randomly selected as blank controls. The strain HGX-24 was inoculated at 10% (*v*/*v*) into each bottle and incubated in a shaking incubator at 30 °C and 180 rpm. Samples were collected every 4 h for a total of 12 time points. The optical density at 600 nm (OD600) was measured using a spectrophotometer (TU-1901, Beijing Purkinje General Instrument Co., Ltd., Beijing, China), with the blank controls used for zero adjustment. Growth curves were plotted with time (h) on the *x*-axis and OD600 on the *y*-axis.

To investigate transcriptional responses under different cadmium conditions, three treatment groups were established, each with three biological replicates:

Group A (control), 10 mL of seed culture was inoculated into 100 mL of fresh LB medium;

Group B (low Cd^2+^ stress), 10 mL of seed culture was inoculated into 100 mL of LB medium supplemented with 175 mg/L Cd^2+^;

Group C (high Cd^2+^ stress), 10 mL of seed culture was inoculated into 100 mL of LB medium supplemented with 350 mg/L Cd^2+^.

All cultures were incubated with shaking at 30 °C and 180 rpm.

### 2.2. Scanning Electron Microscopy and Energy-Dispersive X-Ray Spectroscopy

Take 10 mL of the bacterial cultures from Group A (control) and Group C (350 mg/L Cd^2+^ stress) respectively, at the bacterial concentration reaches approximately 1 × 10^9^ CFU/mL. After centrifugation at 8000 rpm and 4 °C for 5 min, the resulting bacterial pellets were washed three times with 10× PBS buffer.

Samples were prepared for scanning electron microscopy (SEM) (TESCAN, Brno, Czech Republic) according to a standard protocol. The samples were fixed overnight with 2.5% glutaraldehyde at 4 °C, followed by three washes with phosphate buffer (pH 7.2) for 15 min each. Gradient dehydration was then performed using an ascending ethanol series (30%, 50%, 70%, 80%, 90%, and 100%) for 15 min at each concentration, with two changes in 100% ethanol. The dehydrated samples were subsequently replaced with isoamyl acetate for 20 min (twice). Finally, the samples were dried using critical point drying with carbon dioxide and observed under SEM. Energy-dispersive X-ray spectroscopy (EDS) (XFlash 7, Bruker, Billerica, MA, USA) was performed simultaneously to analyze the elemental composition of the bacterial surface.

### 2.3. FTIR Analysis of Strain HGX-24 Before and After Cadmium Treatment

Bacterial cultures (10 mL) from Groups A, B, and C were collected at the stationary growth phase and centrifuged at 8000 rpm for 15 min. The resulting bacterial pellets were washed 3–5 times with sterile deionized water. The washed pellets were then freeze-dried overnight. For Fourier-transform infrared (FTIR) spectroscopy analysis, the freeze-dried samples were mixed with potassium bromide (KBr) at a 1:100 (*w*/*w*) ratio, finely ground, and pressed into transparent pellets using a hydraulic press. The prepared pellets were analyzed using an FTIR spectrometer (Nicolet iS10, Thermo Fisher Scientific, Waltham, MA, USA) to characterize surface functional groups [27].

### 2.4. Transcriptomics Analysis

#### 2.4.1. RNA Extraction, cDNA Library Construction and Illumina Sequencing

After 16 h of incubation, 10 mL of culture from Groups A (control) and C (high Cd^2+^ stress) were collected. The samples were centrifuged at 4000 rpm for 10 min at 4 °C. The resulting bacterial pellets were washed 3–5 times with sterile deionized water and re-centrifuged at 1500 rpm for 15 min at 4 °C. The washed pellets were immediately snap-frozen in liquid nitrogen and stored at −80 °C until further processing. Total RNA was subsequently extracted and its quality assessed. High-quality RNA samples were used for cDNA library construction (insert size 300–400 bp), followed by sequencing on the Illumina HiSeq 2000 platform (Illumina, San Diego, CA, USA).

#### 2.4.2. mRNA Gene Expression Level Analysis

Raw sequencing reads were processed using Fastp (v0.20.1) to remove 3′ adapter sequences and low-quality reads (average Phred quality score < Q20), yielding high-quality clean reads. The reference genome and corresponding gene annotation files were downloaded from public databases. The raw RNA-Seq data generated in this study have been deposited in the NCBI Sequence Read Archive (SRA) database under BioProject accession number PRJNA1452542 and BioSample accession numbers SAMN57245509–SAMN57245514. Bowtie2 (v2.5.1) was employed to build an index of the reference genome and to align the clean reads to the genome. HTSeq (v0.6.1p2) was then used to quantify raw read counts for each gene, representing the unnormalized expression levels [28]. Gene expression levels were normalized using the fragments per kilobase of transcript per million mapped reads (FPKM) method to enable cross-gene and cross-sample comparisons. For reference-based transcriptomes, genes with FPKM > 1 were considered expressed. To assess sample similarity and the overall quality of the transcriptomic data, principal component analysis (PCA) was performed on the FPKM values of all expressed genes using the R package prcomp (the prcomp function in R [v4.5.3]).

#### 2.4.3. Differential Expression Gene Analysis

Differential gene expression analysis was performed using DESeq (v1.46.0). Genes were considered differentially expressed if they met the following criteria: |log_2_(fold change)| > 1 and adjusted *p*-value < 0.05 and |log_2_(fold change)| < 1 and adjusted *p*-value < 0.05. Hierarchical clustering was conducted to visualize expression patterns of the differentially expressed genes (DEGs) across treatment groups. Genes and samples were clustered based on expression correlation. Two-way hierarchical clustering of DEGs and samples was performed using the R package pheatmap (v1.0.13), with Euclidean distance as the dissimilarity measure and complete linkage as the agglomeration method.

Gene Ontology (GO) enrichment analysis of DEGs was performed using the topGO package (v2.46.0). The distribution of gene annotations across GO terms was statistically evaluated, and significantly enriched GO terms were identified based on the hypergeometric distribution test (*p* < 0.05). Enriched terms were annotated to reveal the biological functions, molecular processes, and cellular components associated with the DEGs under cadmium stress.

#### 2.4.4. The qPCR Analysis of Functional Genes in HGX-24

To validate the reliability of the RNA-seq results, ten DEGs were randomly selected based on the transcriptome data. Real-time quantitative PCR (qPCR) was performed using 16S rRNA as the internal reference gene (coefficient of variation < 5%). Each qPCR reaction included three biological replicates and three technical replicates. The expression levels determined by qPCR were compared with those from RNA-seq to assess correlation [29]. The selected genes and their corresponding primer sequences are listed in Table 1.

### 2.5. Synergistic Remediation of Cadmium-Contaminated Soil by Corn and HGX-24

The potting soil was prepared by mixing campus soil and nutrient-rich soil at a 2:3 (*v*/*v*) ratio. Impurities such as stones and *Z. mays* debris were removed, and the mixture was air-dried and sterilized before being dispensed into pots at 2 kg per pot. The pot experiment consisted of six treatments arranged in a completely randomized design, with three replicates per treatment:

Group 1: No Cd addition, no HGX-24 inoculation (control).

Group 2: No Cd addition, HGX-24 inoculation.

Group 3: 175 mg/L Cd addition, no HGX-24 inoculation.

Group 4: 175 mg/L Cd addition, HGX-24 inoculation.

Group 5: 350 mg/L Cd addition, no HGX-24 inoculation.

Group 6: 350 mg/L Cd addition, HGX-24 inoculation.

For preparation of the HGX-24 inoculum, glycerol-preserved strain HGX-24 was streaked onto LB agar plates and incubated inverted at 30 °C. A single colony was inoculated into LB broth and cultured in a shaking incubator (180 rpm, 30 °C) for 16 h. The culture was centrifuged at 5000 rpm for 10 min at 4 °C, and the pellet was washed at least three times with sterile deionized water and resuspended. The optical density was measured using a UV spectrophotometer, and the suspension was adjusted to approximately 1 × 10^9^ CFU/mL before storage at 4 °C until use.

Cadmium was added to the soil as CdCl_2_ solution to achieve the target concentrations. After a 7-day equilibration period, an equal volume of HGX-24 suspension was applied to the inoculated groups, while an equal volume of sterile distilled water was added to the non-inoculated groups. Following another 7-day equilibration, five pre-germinated (overnight-soaked) *Z. mays* seeds were sown per pot at a depth of 4–5 cm. Soil moisture was maintained throughout germination and growth, and *Z. mays* development was monitored regularly.

After 60 days of growth, *Z. mays* plants were harvested. Roots and shoots were carefully rinsed with distilled water, and plant height and fresh biomass were recorded. CAT activity was determined using the ammonium molybdate method [30], POD activity by the colorimetric method [31], and SOD activity by the extraction method [32].

### 2.6. Quantification of Cadmium Ion Concentration in Z. mays Plants

*Z. mays* plants grown for 60 days under different treatment conditions were harvested, thoroughly washed, and separated into aboveground and belowground parts. The *Z. mays* tissues were oven-dried at 60 °C to constant weight, ground into powder, and passed through an 80-mesh sieve for subsequent analysis. The dried *Z. mays* tissues were digested using HNO_3_-HClO_4_ (4:1, *v*/*v*) wet digestion method. The cadmium (Cd) content in each part of the *Z. mays* was determined by inductively coupled plasma mass spectrometry (ICP-MS) (7700x, Agilent Technologies, Santa Clara, CA, USA) after constructing a standard curve.

## 3. Results and Analysis

### 3.1. The Growth Curves of Strain HGX-24 at Different Cadmium Concentrations

**Figure 1:**
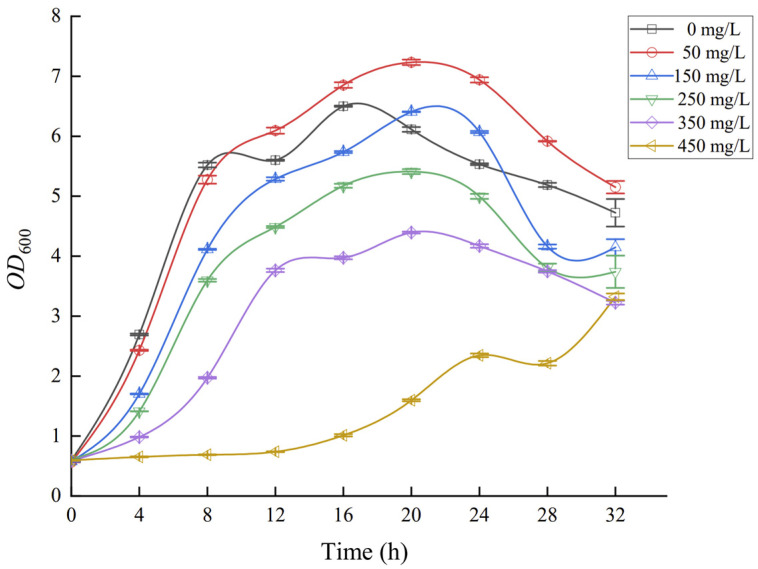
Growth curve of strain HGX-24.

Figure 1 shows the growth trends of strain HGX-24 under different initial cadmium (Cd^2+^) concentrations of 0, 50, 150, 250, 350, and 450 mg/L. As shown in Figure 1, at 0 mg/L Cd^2+^, strain HGX-24 exhibited a short lag phase and reached the late exponential phase after approximately 16 h, with a cell concentration of 6.5 × 10^9^ CFU/mL. This indicates that the strain grew well in the absence of cadmium, with low cultivation cost and ease of culture. Under low cadmium concentration (50 mg/L), the growth of strain HGX-24 was slightly promoted. However, as the cadmium concentration in the medium increased, the exponential phase was progressively delayed and the final cell concentration gradually decreased. Further analysis revealed that the strain could tolerate a maximum cadmium concentration of 350 mg/L while maintaining stable genetic performance.

### 3.2. SEM-EDS Analysis of Strain HGX-24 Before and After Cadmium Treatment

**Figure 2:**
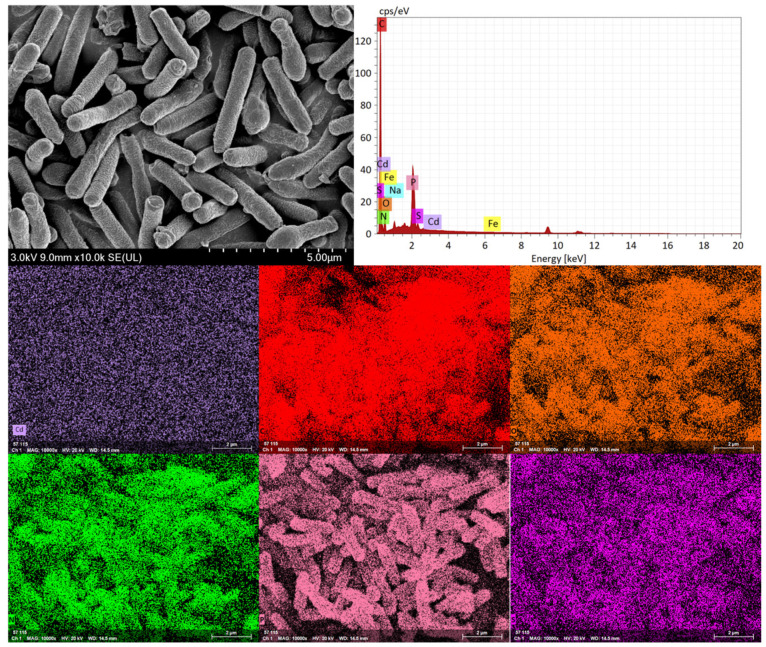
Scanning electron micrographs of strain HGX-24 showing cell morphology and surface elemental distribution in the absence of cadmium.

**Figure 3:**
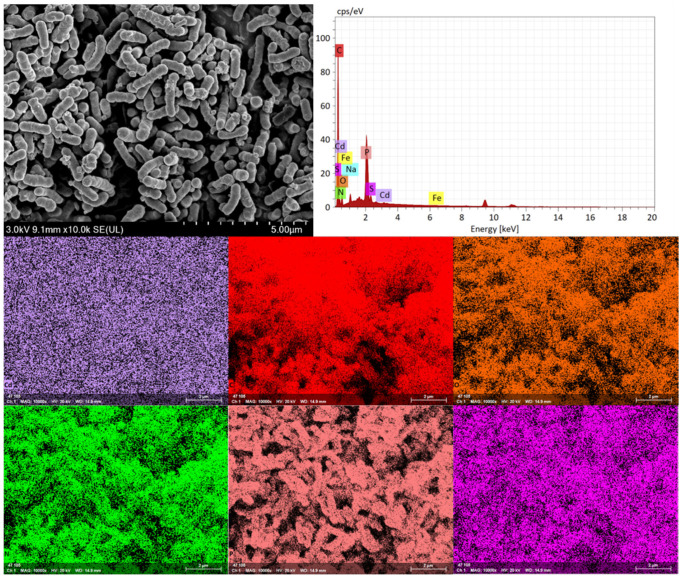
Scanning electron micrographs of strain HGX-24 showing cell morphology and surface elemental distribution in the presence of 350 mg/L cadmium ions.

SEM was used to examine morphological changes in HGX-24 cells from Group A (control) and Group C (350 mg/L Cd^2+^ stress). As shown in Figure 2 and Figure 3, control cells (Group A) appeared as typical short rods with lengths of 3.2–4.5 μm and diameters of 0.7–1.0 μm, exhibiting smooth and uniform surfaces. In contrast, cells exposed to high Cd^2+^ (Group C) displayed irregular rod shapes, with reduced dimensions (lengths 1.0–1.8 μm, widths 0.3–0.8 μm) and pronounced surface abnormalities, including bamboo-like segmentation, obvious shrinkage, bending, and partial rupture. Concurrent EDS analysis revealed significant elemental changes in the stressed cells compared to the control: decreased carbon (C) and oxygen (O) contents, accompanied by markedly increased levels of sulfur (S), phosphorus (P), nitrogen (N), cadmium (Cd), and other elements.

### 3.3. FTIR Analysis of Strain HGX-24 Under Different Cadmium Treatments

**Figure 4:**
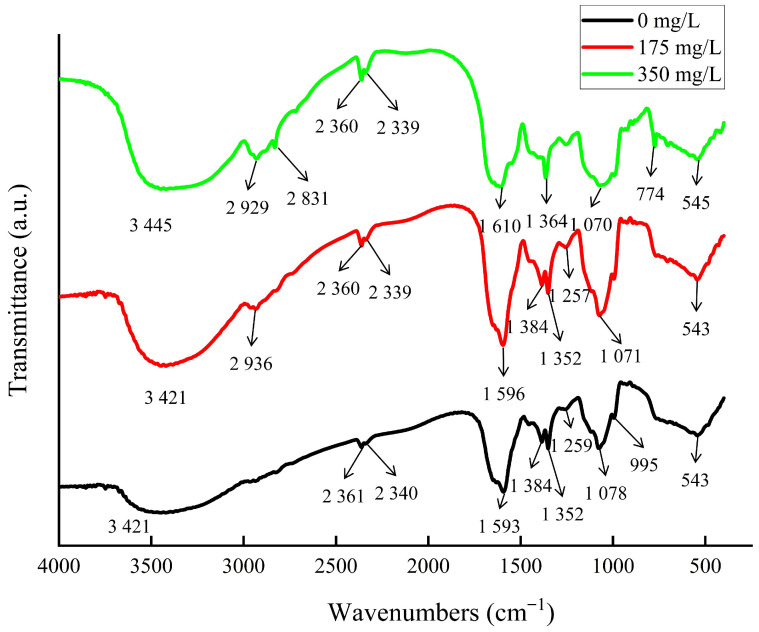
FTIR infrared spectrum analysis of HGX-24.

Several surface functional groups on strain HGX-24 play a critical role in the adsorption of cadmium ions. As shown in Figure 4, the FTIR spectrum of the control cells (Group A, no Cd^2+^) displayed characteristic absorption peaks spanning the range of 500–4000 cm^−1^. With increasing Cd^2+^ concentration (Groups B and C), distinct shifts in peak positions and intensities were observed, indicating direct involvement of these functional groups in Cd^2+^ binding.

The broad absorption band centered at 3421 cm^−1^ is assigned to the stretching vibrations of O−H and N−H groups. With increasing Cd^2+^ concentration, this peak broadened and underwent a blue shift (to lower wavenumbers), indicating that hydroxyl (−OH) and amino (−NH) groups on the cell surface actively participate in Cd^2+^ coordination through their stretching vibrations.

New or intensified absorption peaks emerged at 2929 cm^−1^, 2831 cm^−1^, and 774 cm^−1^ with increasing Cd^2+^ concentration. These peaks are primarily associated with C-H stretching vibrations and O-H bending vibrations, providing evidence that carboxyl groups (−COOH) play an active role in the adsorption and accumulation of cadmium ions.

The amide II band, located at approximately 1593 cm^−1^ and attributed to the in-plane deformation vibration of N-H in protein amide groups, exhibited a blue shift of 3 cm^−1^ in Group B and 17 cm^−1^ in Group C relative to the control (Group A). This shift was accompanied by a progressive decrease in peak intensity, providing clear evidence that the amide groups (−CONH−) participate in Cd^2+^ coordination and binding.

The amide III band, centered at approximately 1259 cm^−1^ and arising from coupled C=O stretching and N-H bending vibrations in protein amide groups, exhibited a red shift of 2 cm^−1^ in Group B and 5 cm^−1^ in Group C relative to the control (Group A). This shift provides evidence that the amide groups (−CONH−) actively participate in the coordination and enrichment of cadmium ions.

The absorption peak at 1078 cm^−1^, arising from the combined contributions of phosphate group vibrations and C-N stretching in amino groups, shifted to higher wavenumbers (red shift) by approximately 7 cm^−1^ in Group B and 8 cm^−1^ in Group C compared to the control (Group A), accompanied by a progressive decrease in peak intensity. These spectral changes provide strong evidence that phosphate (PO_4_^3−^) and amino (−NH_2_) groups on the cell wall of strain HGX-24 are actively involved in the adsorption of cadmium ions.

The absorption peaks at 1384 cm^−1^ and 1352 cm^−1^, attributed to −CH bending vibrations, showed notable changes under Cd^2+^ stress. In Group C (high Cd^2+^), the 1384 cm^−1^ peak completely disappeared, while the 1352 cm^−1^ peak exhibited a blue shift of approximately 12 cm^−1^ relative to the control (Group A).

Additionally, the low-wavenumber peak at 543 cm^−1^, associated with in-plane bending vibrations of C−C=O and C−X (X = halogen), underwent a gradual blue shift of 2 cm^−1^ with increasing Cd^2+^ concentration.

Collectively, these FTIR spectral changes demonstrate that multiple functional groups on the cell wall and extracellular matrix of strain HGX-24—including carboxyl (−COOH), amide (−CONH−), amino (−NH), hydroxyl (−OH), and carbonyl (>C=O) groups—play key roles in the direct adsorption and immobilization of cadmium ions.

### 3.4. Sequencing Data Processing and Alignment Statistics

As shown in Table 2, raw sequencing of the control group (Group A; Samples 1–3) generated 15,471,148, 19,983,576, and 16,029,654 reads, respectively. After quality filtering, 15,236,368, 19,681,238, and 15,800,462 high-quality clean reads were retained, corresponding to 98.48%, 98.49%, and 98.57% of the total reads, respectively. For the high-Cd^2+^ stress group (Group C; Samples 4–6), raw read counts were 17,565,868, 17,580,244 and 16,191,316, with 17,334,048, 17,340,628 and 15,942,834 clean reads retained after filtering, accounting for 98.68%, 98.64%, and 98.47% of the raw data, respectively.

Clean reads were aligned to the reference genome (GCF_027920505.1_ASM2792050v1-genomic.fna) using SOAPaligner/soap2 (v2.21; BGI-Shenzhen, Shenzhen, China). As detailed in Table 3, the overall mapping rates for the six samples ranged from 86.72% to 90.85%, with specific values of 87.09%, 87.11%, 86.72%, 90.57%, 90.85%, and 90.39%. The proportions of reads mapping to multiple genomic positions were 40.36%, 40.29%, 40.67%, 35.97%, 35.83%, and 34.78%, respectively.

According to Table 4, the percentages of reads aligned to annotated gene regions (relative to total reads) were 96.93%, 97.09%, 97.00%, 93.46%, 93.53%, and 93.95%, respectively. The proportions of reads mapping to rRNA, tRNA, sRNA, and ncRNA were very low across all samples, confirming effective removal of non-coding RNAs during library preparation and preprocessing.

Collectively, these alignment statistics—including high overall mapping rates, low multi-mapping proportions, strong gene-region coverage, and minimal residual non-coding RNA—demonstrate excellent sequencing data quality, fully meeting the requirements for downstream differential expression analysis and functional annotation.

### 3.5. Analysis of mRNA Gene Expression Levels

**Figure 5:**
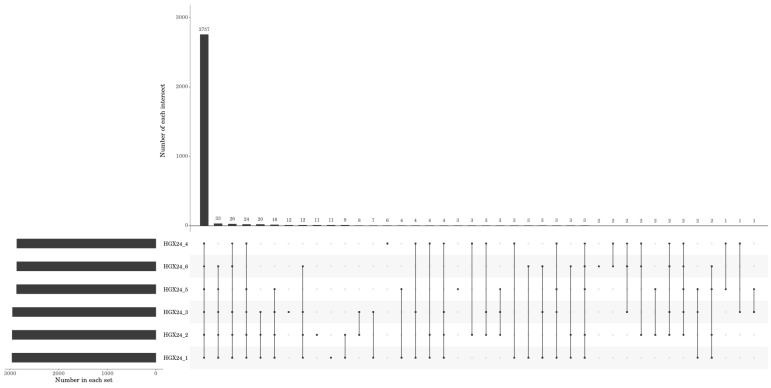
Upset plot of mRNAs identified in each sample.

**Figure 6:**
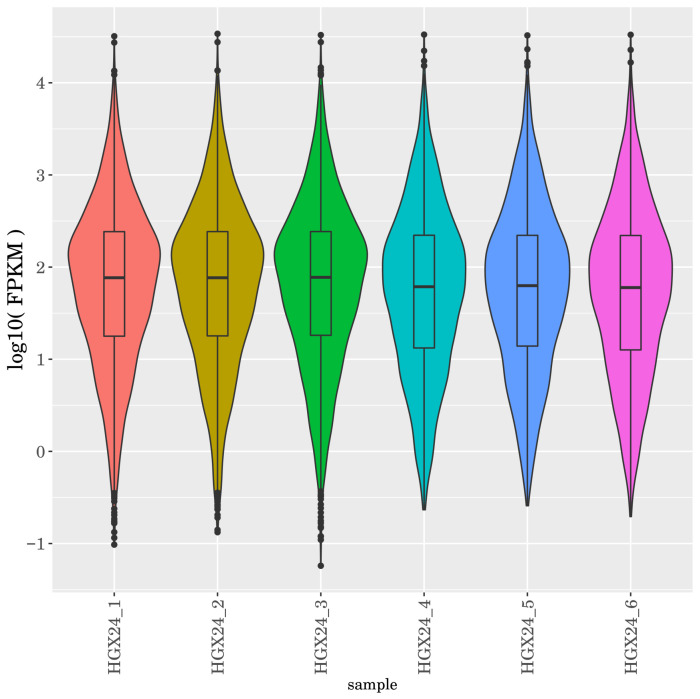
FPKM density distribution statistics.

**Figure 7:**
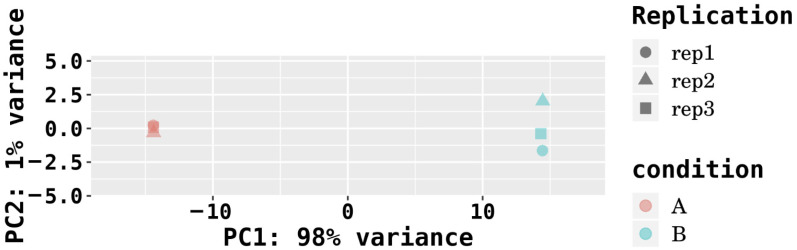
PCA.

The identified mRNAs in each sample were compiled based on their calculated expression levels. Common and unique mRNAs across the six samples were visualized using an UpSet plot (Figure 5), highlighting the overlap and specificity of transcript detection among the control and Cd-treated groups. The FPKM density boxplot (Figure 6) revealed that genes with moderate expression levels constituted the predominant proportion, forming the main peak in the distribution, while genes with extremely low or high expression levels represented smaller fractions. These patterns indicate that the transcriptomes of the six samples are evenly distributed and exhibit highly similar overall expression profiles. Principal component analysis (PCA) was performed to reveal the similarity among samples, where the distance between samples is negatively correlated with their similarity. As shown in Figure 7, the three samples in Group A (Samples 1, 2, and 3) exhibited high similarity, and the three samples in Group B (Samples 4, 5, and 6) also clustered closely together. This pattern is consistent with the experimental grouping.

### 3.6. Differential Analysis of Gene Expression

**Figure 8:**
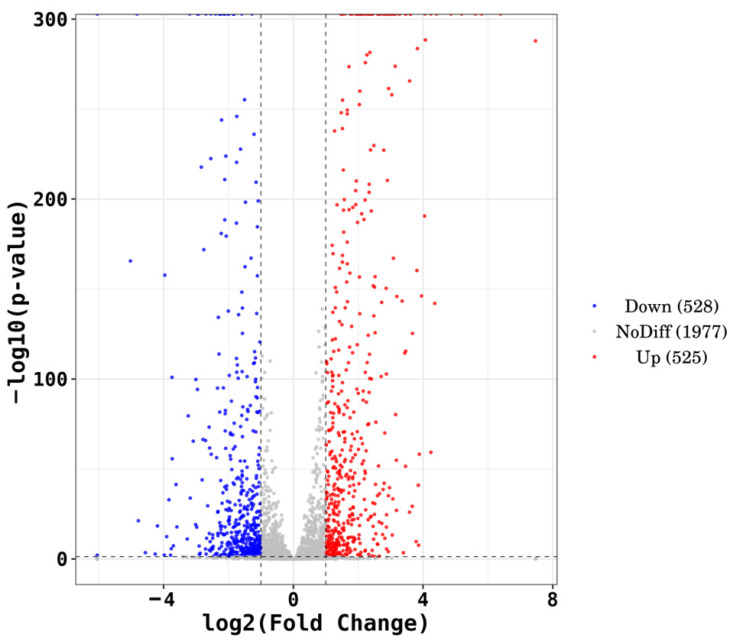
Volcano plot of differentially expressed genes.

The volcano plot (Figure 8) was constructed using thresholds of |log_2_(fold change)| = 1 (two vertical dashed lines) and *p* = 0.05 (one horizontal dashed line). Red dots represent significantly upregulated genes (log_2_FC ≥ 1, *p* < 0.05), blue dots represent significantly downregulated genes (log_2_FC ≤ −1, *p* < 0.05), and gray dots indicate genes with no significant differential expression. Compared with the control group (Group A), a total of 1053 DEGs were identified in the high-Cd^2+^ stress group (Group C), comprising 525 upregulated genes and 528 downregulated genes. The detailed information of these 1053 DEGs is provided in Supplementary Table S1. The number of downregulated genes was slightly higher than that of upregulated genes.

### 3.7. GO Functional Analysis of Differentially Expressed Genes

GO enrichment analysis was performed on the DEGs using the topGO package to identify significantly over-represented GO terms and to provide functional classification of the DEGs. In the reference genome, a total of 3030 genes were mapped, of which 874 (28.84%) received GO annotations. Among the 1053 DEGs identified between the control (Group A) and high-Cd^2+^ stress (Group C) groups, 283 DEGs (22.70%) were successfully annotated with GO terms.

**Figure 9:**
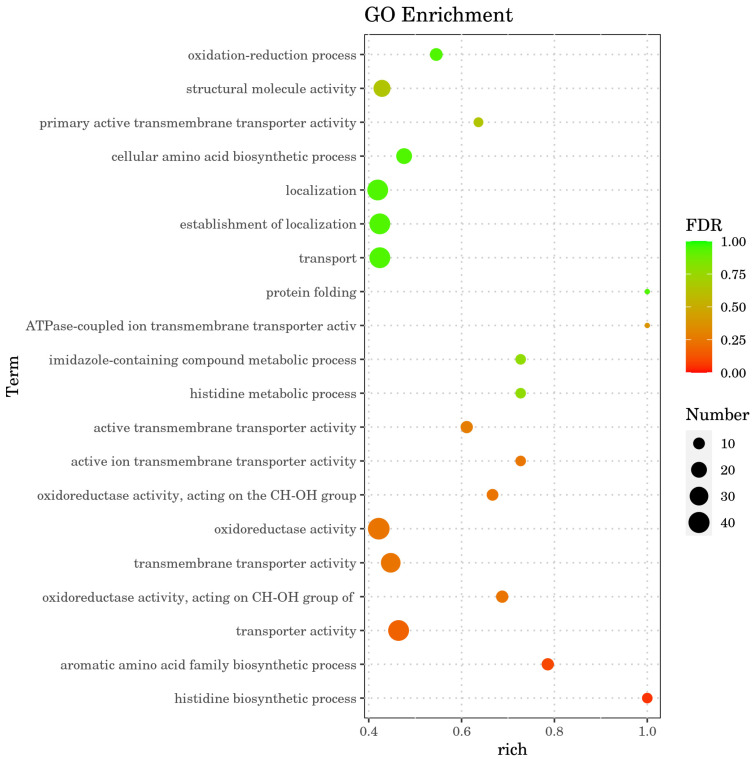
Bubble chart of GO enrichment analysis.

GO enrichment analysis of the differentially expressed genes (DEGs) revealed that, relative to the control group (Group A), the significantly upregulated DEGs in the high-Cd^2+^ stress group (Group C) were annotated with 756 GO terms, while the significantly downregulated DEGs were annotated with 650 GO terms. The top 20 significantly enriched GO terms (*p* < 0.05) were selected for detailed statistical analysis (Table 5). As illustrated in Figure 9, the most highly enriched GO terms included histidine biosynthetic process, aromatic amino acid family biosynthetic process, transporter activity, oxidoreductase activity, and transmembrane transporter activity. These enrichment patterns suggest that the cadmium resistance mechanism of strain HGX-24 is closely associated with these biological processes and molecular functions.

### 3.8. KEGG Pathway Analysis

Annotation against the KEGG database revealed that 1368 of the 3030 mapped genes (45.15%) were assigned to KEGG pathways. Among the 1053 DEGs identified—525 upregulated and 528 downregulated—271 upregulated DEGs (51.62%) and 221 downregulated DEGs (41.86%) were successfully annotated to KEGG pathways.

**Figure 10:**
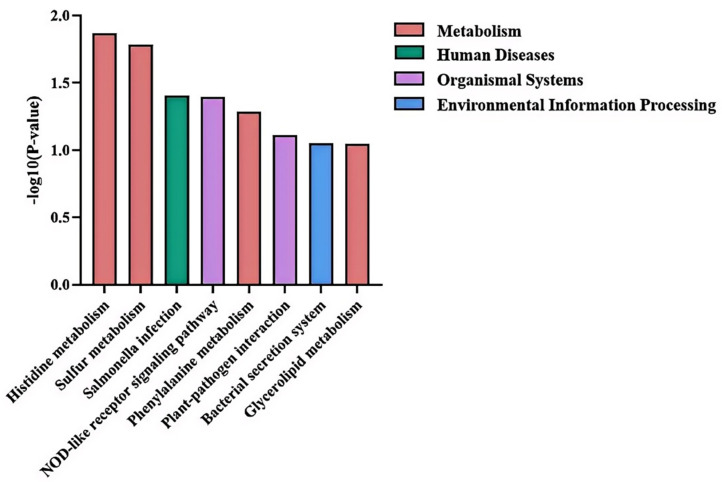
Histogram of KEGG pathway enrichment analysis.

KEGG pathway enrichment analysis showed significant over-representation (*p* < 0.05) of DEGs in several key pathways, including histidine metabolism, sulfur metabolism, Salmonella infection, phenylalanine metabolism, NOD-like receptor signaling pathway, plant-pathogen interaction, bacterial secretion system, and glycolipid metabolism (detailed in Table 6 and Figure 10).

### 3.9. The qPCR Verification

**Figure 11:**
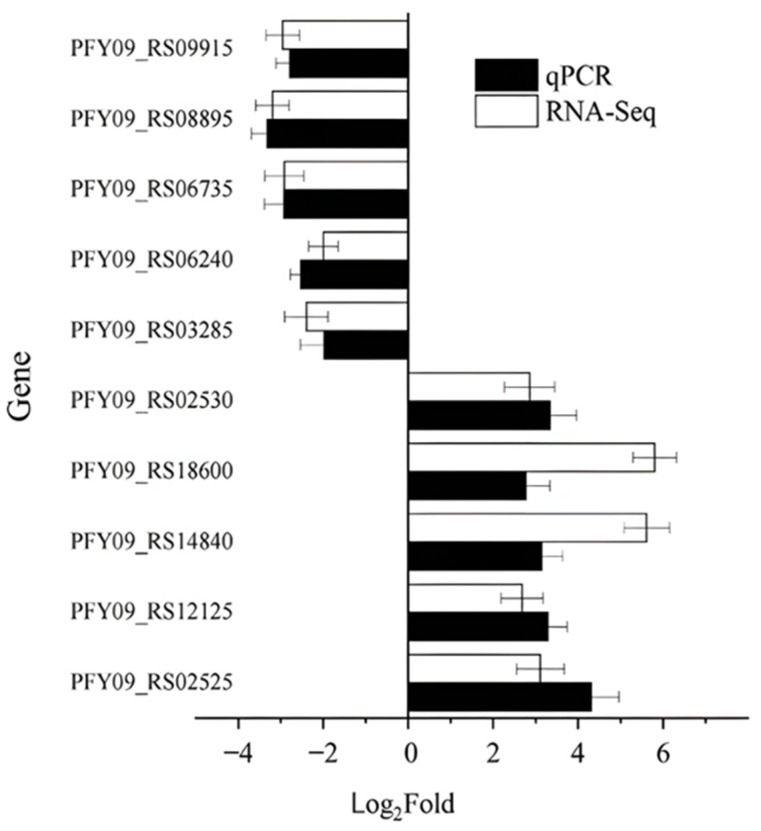
The qPCR validation of differentially expressed genes.

The 10 genes selected for qPCR validation were randomly chosen from the significantly differentially expressed genes (DEGs) to independently verify the reliability of the RNA-seq results. As shown in Figure 11, the qPCR expression levels of these genes were highly consistent with the RNA-seq data (Pearson r = 0.931, *p* < 0.001, *n* = 10), confirming the biological reliability and reproducibility of the transcriptomic dataset.

### 3.10. Effects of HGX-24 on Z. mays Growth Under Cadmium Stress

**Figure 12:**
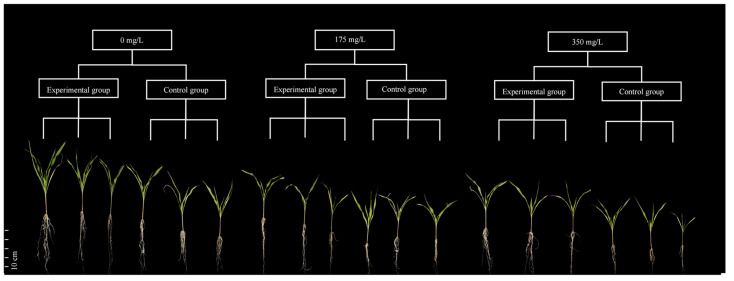
Growth morphology of *Z. mays* under Cd concentration gradients. 0 mg/L Cd Control group: Group 1 (no HGX-24 inoculation); Experimental group: Group 2 (HGX-24 inoculation). 175 mg/L Cd Control group: Group 3 (no HGX-24 inoculation); Experimental groups: Group 4 (HGX-24 inoculation). 350 mg/L Cd Control group: Group 5 (no HGX-24 inoculation); Experimental groups: Group 6 (HGX-24 inoculation).

Pot experiments showed that after sixty days of cultivation, with the increase of cadmium concentration in the soil, the plant height of corn presented a significant downward trend. However, under identical Cd levels, plants inoculated with HGX-24 strain consistently showed superior growth performance compared to non-inoculated controls (Figure 12). These observations indicate that HGX-24 reduced the cadmium-induced growth suppression in *Z. mays*.

**Figure 13:**
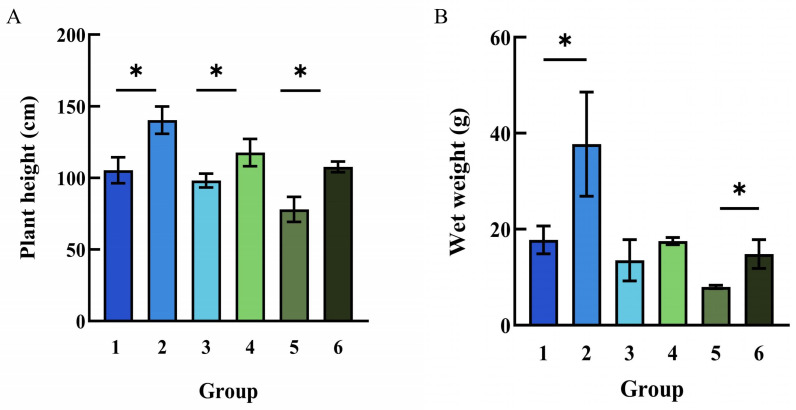
Changes in plant height and wet weight of *Z. mays* under different cadmium concentration. ((**A**). Plant height; (**B**). Wet weight. *: *p* < 0.05). Control group: Group 1 (no HGX-24 inoculation), Group 3 (175 mg/L Cd, no HGX-24 inoculation), Group 5 (350 mg/L Cd, no HGX-24 inoculation); Experimental group: Group 2 (HGX-24 inoculation), Group 4 (175 mg/L Cd, HGX-24 inoculation), Group 6 (350 mg/L Cd, HGX-24 inoculation).

To statistically confirm these differences, plant height and fresh biomass were analyzed using *t*-tests after the *Z. mays* plants had been grown for 60 days (Figure 13). In the absence of HGX-24 inoculation, plant height in Group 3 decreased by approximately 14.88% and fresh biomass by 23.82% compared with Group 1. In Group 5, plant height was reduced by 32.37% and fresh biomass by 55.07%, confirming strong cadmium-induced growth inhibition.

In contrast, inoculation with HGX-24 significantly promoted growth even in the absence of Cd stress: compared with Group 1, Group 2 showed increases of 21.68% in plant height and 112.33% in fresh biomass, indicating that the strain is non-toxic and exerts a growth-promoting effect. Under Cd stress, HGX-24 inoculation markedly alleviated inhibition: compared with Group 3, Group 4 exhibited 19.86% higher plant height and 29.71% higher fresh biomass; compared with Group 5, Group 6 showed 38.04% higher plant height and 85.71% higher fresh biomass. These results demonstrate that strain HGX-24 effectively counteracts cadmium-induced growth suppression in *Z. mays*.

### 3.11. Changes in the Activity of Redox Enzymes in Z. mays Under Different Treatment Conditions

**Figure 14:**
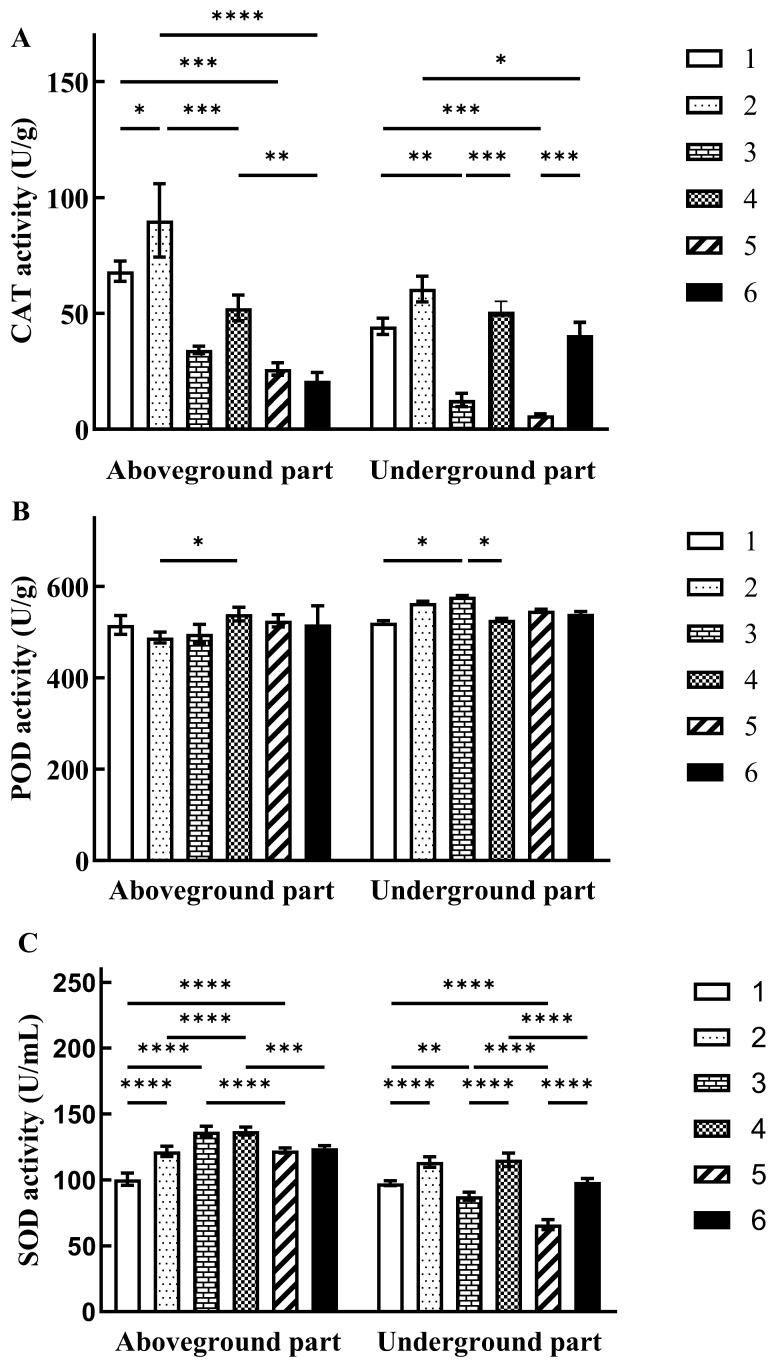
Activities of three oxidoreductases in aboveground and underground parts of *Z. mays* under different Cd concentrations. ((**A**). CAT activity; (**B**). POD activity; (**C**). SOD activity. *: *p* < 0.05; **: *p* < 0.01; ***: *p* < 0.001; ****: *p* < 0.0001). Control group: Group 1 (no HGX-24 inoculation), Group 3 (175 mg/L Cd, no HGX-24 inoculation), Group 5 (350 mg/L Cd, no HGX-24 inoculation); Experimental group: Group 2 (HGX-24 inoculation), Group 4 (175 mg/L Cd, HGX-24 inoculation), Group 6 (350 mg/L Cd, HGX-24 inoculation).

To further investigate the impact of strain HGX-24 on antioxidant defense in *Z. mays* under cadmium stress, the activities of three key redox enzymes—CAT, POD, and SOD—were measured in both aboveground (shoots) and underground (roots) tissues. As illustrated in Figure 14, *t*-test analysis revealed that, across all cadmium concentrations, enzyme activities were consistently higher in *Z. mays* plants inoculated with HGX-24 compared to non-inoculated controls. These results indicate that HGX-24 enhances *Z. mays* tolerance to Cd-induced oxidative stress by elevating CAT, POD and SOD activities.

Specifically, compared with Group 1, Group 2 exhibited a 21.01% increase in SOD activity in shoots, a 16.58% increase in CAT activity in roots, an 8.25% increase in POD activity in roots, and a 36.70% increase in SOD activity in roots. Under low Cd stress, Group 4 showed a 52.28% increase in CAT activity and an 8.60% increase in POD activity in shoots, a substantial 303.23% increase in CAT activity in roots, a slight 8.24% decrease in POD activity in roots, and a 31.50% increase in SOD activity in roots compared with Group 3. Under high Cd stress, Group 6 displayed a remarkable 56.67% increase in CAT activity and a 49.57% increase in SOD activity in roots compared with Group 5.

Overall, these findings demonstrate that inoculation with HGX-24 strain significantly enhances the activities of antioxidant enzymes in *Z. mays*, thereby effectively alleviating Cd-induced oxidative damage, mitigating reactive oxygen species accumulation, and supporting improved metabolism and growth under cadmium stress.

### 3.12. The Variation of Cadmium Ion Content in Corn Under Different Treatment Conditions

**Figure 15:**
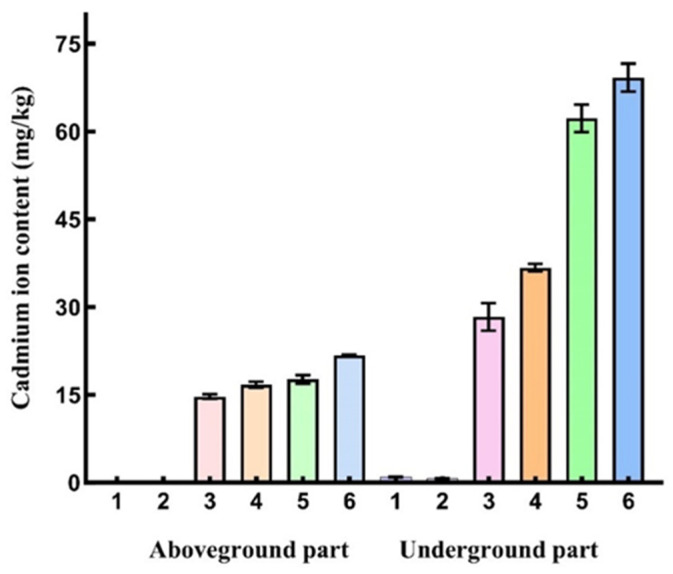
Changes in cadmium ion content in *Z. mays* under different treatment conditions. Control group: Group 1 (no HGX-24 inoculation), Group 3 (175 mg/L Cd, no HGX-24 inoculation), Group 5 (350 mg/L Cd, no HGX-24 inoculation); Experimental group: Group 2 (HGX-24 inoculation), Group 4 (175 mg/L Cd, HGX-24 inoculation), Group 6 (350 mg/L Cd, HGX-24 inoculation).

The cadmium (Cd) contents in the aboveground and belowground parts of *Z. mays* under different treatment groups were determined. As shown in Figure 15, the results indicated that Cd accumulation increased significantly in both aboveground and belowground parts of *Z. mays* with increasing cadmium pollution levels. This accumulation was particularly pronounced in the belowground parts. When the exogenous Cd concentration reached 350 mg/L, the Cd content in the belowground parts of *Z. mays* in Group 5 was approximately 62.26 mg/kg higher than that in the control Group 1 and 33.93 mg/kg higher than that in Group 3. Similarly, the Cd content in the belowground parts of *Z. mays* in Group 6 was approximately 69.20 mg/kg higher than that in Group 2 and 32.46 mg/kg higher than that in Group 4. In all treatment groups, the Cd content in the belowground parts were significantly higher than that in the aboveground parts, confirming the cadmium accumulation pattern in *Z. mays* characterized by root absorption followed by partial translocation. Furthermore, the Cd contents in *Z. mays* inoculated with strain HGX-24 (Groups 2, 4, and 6) were higher than those in the non-inoculated groups (Groups 1, 3, and 5). This difference was more evident in the belowground parts, indicating that inoculation with strain HGX-24 enhanced Cd uptake and accumulation in *Z. mays*, particularly in the roots, and played a promotional role in the absorption and translocation of cadmium by *Z. mays*.

## 4. Discussion

Cadmium is a potentially toxic metal that exerts concentration-dependent effects on environmental microorganisms. At low concentrations, Cd can positively influence microbial processes, including protein synthesis, enzyme structural stability, and redox reactions. At high concentrations, however, Cd disrupts DNA and RNA synthesis, alters enzyme conformations, and directly damages cellular structures, leading to loss of membrane integrity, cell shrinkage, and other morphological abnormalities [33,34]. Current studies have established that microbial resistance to cadmium primarily involves two major strategies: surface adsorption and intracellular accumulation. Extracellular polymeric substances (EPS), complex high-molecular-weight biopolymers secreted during microbial growth and metabolism, are composed mainly of proteins, polysaccharides, and lipids. EPS plays a pivotal role in the adsorption and immobilization of Cd^2+^ ions, serving as the first line of defense against heavy metal toxicity [35].

At lower Cd concentrations, extracellular protein secretion by HGX-24 strain was significantly upregulated, suggesting an adaptive mechanism to mitigate cadmium toxicity. However, at excessively high Cd levels, this secretion was markedly inhibited. Notably, the content of extracellular insoluble polysaccharides exhibited a strong negative correlation with Cd concentration, likely reflecting a regulatory response to cadmium stress. EDS analysis revealed decreased surface C and O contents under Cd exposure, accompanied by substantial increases in N, P, S, and Cd levels. These compositional changes indicate that the strain resists Cd stress by actively modifying its EPS. Furthermore, FTIR analysis demonstrated that negatively charged functional groups on the cell wall including −COOH, −CONH−, −NH, −OH, >C=O, and phosphate groups coordinate with Cd^2+^, thereby facilitating the adsorption and sequestration of cadmium ions.

KEGG pathway enrichment analysis revealed significant activation of several key metabolic and stress-response pathways in strain HGX-24 under cadmium stress, including carbon metabolism (EMP, TCA, PPP), purine metabolism, amino acid metabolism, and sulfur metabolism. Pathways related to defense gene induction, phytoalexin accumulation, miRNA production, hypersensitive response, innate immune signaling, and apoptosis were also upregulated. In contrast, genes encoding membrane fusion proteins were downregulated, which may be part of the strain’s adaptive response to cadmium toxicity.

Cadmium contamination in soil and aquatic environments induces a range of physiological disorders in plants, including impaired photosynthesis, reduced soluble protein content, and diminished activity of antioxidant enzymes. This triggers excessive reactive oxygen species (ROS) production, resulting in oxidative stress [36] and consequent growth retardation, decreased biomass, and lower grain yields. Cadmium stress is frequently associated with ROS accumulation, exacerbating oxidative damage [37].

Antioxidant enzymes, which are predominantly proteinaceous, catalyze essential reactions in microbial responses to abiotic stress. They effectively scavenge ROS, thereby mitigating cadmium toxicity in microbial cells. SOD serves as the primary defense, catalyzing the dismutation of superoxide anions (O_2_^−^) to hydrogen peroxide (H_2_O_2_) and oxygen (O_2_), while CAT and POD subsequently decompose excess H_2_O_2_ [38]. In this study, genes encoding SOD family proteins (e.g., PFY09_RS12790, fabG, and ybjT) were significantly upregulated under cadmium stress. These genes play a critical role in ROS scavenging, alleviating oxidative stress, and protecting membrane lipids from peroxidation.

Analysis of plant height and fresh biomass across treatment groups confirmed that cadmium significantly suppressed growth, whereas inoculation with strain HGX-24 substantially mitigated this inhibition. Notably, even in the absence of cadmium, HGX-24 inoculation promoted superior growth relative to uninoculated controls, indicating a beneficial plant growth-promoting effect. Under cadmium stress, CAT, POD, and SOD activities were markedly elevated in inoculated *Z. mays* plants, suggesting that HGX-24 modulates these antioxidant enzymes to attenuate ROS toxicity and enhance overall *Z. mays* metabolism and performance.

## 5. Conclusions

In this study, a cadmium-resistant strain HGX-24 was successfully isolated. HGX-24 exhibited a strong ability to adsorb cadmium ions through extracellular polymeric substances and functional groups such as −COOH, −CONH−, −NH_2_, −OH, >C=O, and phosphate groups on the cell wall. Additionally, HGX-24 modulated the secretion of extracellular proteins and polysaccharides to counteract the adverse effects of cadmium stress on bacterial growth.

Transcriptome analysis shows that under high-concentration cadmium conditions, HGX-24 can regulate the high expression of genes such as antioxidant defense and ROS scavenging (katG, fabG, ybjT), Fe-S cluster assembly (sufB, sufD), sulfur metabolism (cysAU), amino acid metabolism (hisA, cysD, aspC), phenylacetic acid catabolism (paaC), while downregulating genes such as ribosomal proteins (rplC, rpsC, rpsL, rplA, rplY, rpmC). These differentially expressed genes, particularly the significantly upregulated *fabG* and *ybjT*, play critical roles in ROS scavenging and protection of membrane lipids from peroxidation. Overall, they affect the synthesis and metabolism of membrane transporters (ABC transporters and efflux RND transporters), antioxidant enzymes (SOD, CAT, POD), Fe-S clusters, thioredoxin family proteins, and ribosomal proteins, thereby enabling strain HGX-24 to effectively resist the toxic effects of cadmium.

Through the detection of the activities of redox enzymes in *Z. mays*, it was found that cadmium ions can exacerbate the oxidative stress response in *Z. mays*. However, strain HGX-24 can regulate the activities of redox enzymes in *Z. mays*, alleviate the oxidative stress response, reduce the toxic effects of reactive oxygen species on *Z. mays*, and promote the growth and development of *Z. mays*.

In conclusion, the results of this study provide new insights into the cadmium resistance mechanisms of strain HGX-24, which is of great significance for the microbial remediation of cadmium-contaminated farmland.

## Figures and Tables

**Table 1:** Key genes and the primers.

Gene		Gene Function	Primer
Up regulated gene	PFY09_RS02525 (ywbO)	DsbA family oxidoreductase	5′ ACAAGAAGGCAGACCGAATGG 3′ 3′ TTTGTCTACCCTTCGCCTCG 5′
	PFY09_RS12125 (sufB)	Fe-S cluster assembly protein SufB	5′ CGATTACGAAGATTTCCCACG 3′ 3′ CTAATGCTTCTAAAGGGTTGCC 5′
	PFY09_RS14840 (cadA)	Heavy metal translocating P-type ATPase	5′ CAAGATGCGACTGCTCAAGG 3′ 3′ CGATGCTAACTTTGACGGCTC 5′
	PFY09_RS18600 (tssD)	Type VI secretion system tube protein TssD	5′ ATCACCTTCAACAATCCACGC 3′ 3′ CTGAGCCTGTGAGGTTATGCG 5′
	PFY09_RS02530 (mtnN)	Nucleosidase	5′ AGAAATCTACCGTAACCGTCCAA 3′ 3′ CCTGAACCAAAGTCGGACATCG 5′
Down regulated gene	PFY09_RS03285 (rpsF)	30S ribosomal protein S6	5′ AGCGATTCAAGCGAGACGAG 3′ 3′ TGAGGGCAAGATAGACTCAGC 5′
	PFY09_RS06240 (emrA)	HlyD family secretion protein	5′ AGGCTTCCACAACGGCATAC 3′ 3′ TTACTGGTCTTTACGCATCGC 5′
	PFY09_RS06735 (TK1843)	PfkB family carbohydrate kinase	5′ AATGAACAACCGTCCGAAGC 3′ 3′ ATTAGCATCCAAGCGTTACGG 5′
	PFY09_RS08895 (bepG)	Efflux RND transporter permease subunit	5′ AGAGGTTATGGCTACGATTGGC 3′ 3′ TCAAGGCGGCTCACAAGTTCA 5′
	PFY09_RS09915 (czcC)	TolC family protein	5′ TAATCTCGGAGCAGGGTGGAC 3′ 3′ TTTTCTTACTTTCAACGGAGCG 5′

**Table 2:** Statistics of sequencing data filtering.

Sample		Total Reads	Clean Reads	Clean Reads%
Group A	Sample-1	15,471,148	15,236,368	98.48
	Sample-2	19,983,576	19,681,238	98.49
	Sample-3	16,029,654	15,800,462	98.57
Group C	Sample-4	17,565,868	17,334,048	98.68
	Sample-5	17,580,244	17,340,628	98.64
	Sample-6	16,191,316	15,942,834	98.47

**Table 3:** Statistics of RNA Seq map.

Sample	Group A			Group C		
	Sample-1	Sample-2	Sample-3	Sample-4	Sample-5	Sample-6
Total Mapped Reads	13,269,897	17,144,161	13,702,352	15,699,412	15,754,228	144,111,139
Total Mapped Reads%	87.09	87.11	86.72	90.57	90.85	90.39
Uniquely Mapped Reads	7,914,553	10,236,276	8,129,526	10,052,040	10,109,404	9,398,670
Uniquely Mapped Reads%	59.64	59.71	59.33	64.03	64.17	65.22
Multiple Mapped Reads	5,355,344	6,907,885	5,572,826	5,647,372	5,644,824	5,012,469
Multiple Mapped Reads%	40.36	40.29	40.67	35.97	35.83	34.78
Total Bases (bp)	2,336,143,348	3,017,519,976	2,420,477,754	2,652,446,068	2,654,616,844	2,444,888,716
Clean Data	2,296,931,049	2,968,718,043	2,382,948,975	2,614,412,167	2,615,222,900	2,405,095,300
Clean Data%	98.32	98.38	98.45	98.57	98.52	98.37

**Table 4:** Statistics of the distribution of alignment regions.

Sample	Group A			Group C		
	Sample-1	Sample-2	Sample-3	Sample-4	Sample-5	Sample-6
Total Mapped Reads	13,269,897	17,144,161	13,702,352	15,699,412	15,754,228	14,411,139
Inter Gene	407,072	499,119	411,447	1,026,129	1,019,032	871,842
(%)	3.07	2.91	3.00	6.54	6.47	6.05
Gene	12,862,825	16,645,042	13,290,905	14,673,283	14,735,196	13,539,297
(%)	96.83	97.09	97.00	93.46	93.53	93.95
mRNA	10,950,728	14,230,275	11,329,661	7,910,698	7,759,847	7,181,279
(%)	85.13	85.49	85.24	53.91	52.66	53.04
rRNA	586,853	709,604	633,353	691,192	733,085	546,027
(%)	4.56	4.26	4.77	4.71	4.98	4.03
tRNA	24,622	32,870	24,589	22,374	23,557	21,675
(%)	0.19	0.20	0.19	0.15	0.16	0.16
sRNA	1,175,012	1,505,025	1,171,128	5,800,999	5,973,876	5,556,654
(%)	9.13	9.04	8.81	39.53	40.54	41.04
ncRNA	0	0	0	0	0	0
(%)	0.00	0.00	0.00	0.00	0.00	0.00

**Table 5:** GO enrichment analysis.

Category	GO.ID	Term	Up EDGs	Down EDGs	Total	*p*-Value	FDR
BP	GO:0000105	Histidine biosynthetic process	8	0	8	0.000088817	0.048938097
BP	GO:0009073	Aromatic amino acid family Biosynthetic process	10	1	14	0.000348814	0.096098149
MF	GO:0005215	Transporter activity	15	24	84	0.000409577	0.169155342
MF	GO:0016614	Oxidoreductase activity, acting on CH-OH group of donors	7	4	16	0.001202803	0.240990367
MF	GO:0022857	Transmembrane transporter activity	12	22	76	0.002274738	0.240990367
MF	GO:0016491	Oxidoreductase activity	32	11	102	0.00253307	0.240990367
MF	GO:0016616	Oxidoreductase activity, acting on the CH-OH group of donors, NAD or NADP as acceptor	6	4	15	0.002917559	0.240990367
MF	GO:0022853	Active ion transmembrane transporter activity	4	4	11	0.003587239	0.246921645
MF	GO:0022804	Active transmembrane transporter activity	4	7	18	0.004805396	0.283518357
BP	GO:0006547	Histidine metabolic process	8	0	11	0.005650964	0.77842034
BP	GO:0052803	Imidazole-containing compound metabolic process	8	0	11	0.005650964	0.77842034
MF	GO:0042625	ATPase-coupled ion transmembrane transporter activity	1	3	4	0.0074162	0.382861341
BP	GO:0006457	Protein folding	3	1	4	0.009658337	0.94004502
BP	GO:0006810	Transport	15	24	92	0.01217853	0.94004502
BP	GO:0051234	Establishment of localization	15	24	92	0.01217853	0.94004502
BP	GO:0051179	Localization	15	24	93	0.015040995	0.94004502
BP	GO:0008652	Cellular amino acid biosynthetic process	12	8	42	0.017949859	0.94004502
MF	GO:0015399	Primary active transmembrane transporter activity	1	6	11	0.018898862	0.643849206
MF	GO:0005198	Structural molecule activity	1	23	56	0.019212819	0.643849206
BP	GO:0055114	Oxidation-reduction process	12	0	22	0.019267446	0.94004502
BP	GO:0006547	Histidine metabolic process	8	0	11	0.005650964	0.77842034
BP	GO:0052803	Imidazole-containing compound metabolic process	8	0	11	0.005650964	0.77842034

**Table 6:** KEGG enrichment analysis.

Pathway ID	Pathway	Up Number	Down Number	Total Number	*p*-Value	FDR
ko00340	Histidine metabolism	9	0	14	0.013574095	0.873039157
ko00920	Sulfur metabolism	2	4	8	0.016472437	0.873039157
ko05132	Salmonella infection	4	1	7	0.039406785	0.976650965
ko04621	NOD-like receptor signaling pathway	3	1	5	0.040303183	0.976650965
ko00360	Phenylalanine metabolism	8	2	19	0.051907224	0.976650965
ko04626	Plant-pathogen interaction	3	2	8	0.077630699	0.976650965
ko03070	Bacterial secretion system	2	7	18	0.089668682	0.976650965
ko00561	Glycerolipid metabolism	3	1	6	0.090315998	0.976650965

## Data Availability

The original contributions presented in this study are included in the article/Supplementary Material. Further inquiries can be directed to the corresponding author(s).

## Supplementary Materials

The following supporting information can be downloaded at https://www.mdpi.com/article/10.3390/microorganisms14050957/s1: Table S1: 1053 differentially expressed genes (DEGs) with log_2_(fold change) and −log_10_(padj).
