# Supplementary material for: Strand-Specific RNA-Seq Analysis of the Chryseobacterium sp. HGX-24 Transcriptome in Response to Cadmium Stress
Source: Microorganisms. 2026 Apr 23;14(5):957. doi: 10.3390/microorganisms14050957 (PMC13209784; doi:10.3390/microorganisms14050957)
Supplement: Supplementary file 1 [file microorganisms-14-00957-s001.zip › microorganisms-4210447-supplementary.pdf]

id	log2FoldChange	pval	padj	-log10(padj)	regulation
gene-PFY09_RS00285	4.237426302	0	0	300	up
gene-PFY09_RS01815	2.88884985	0	0	300	up
gene-PFY09_RS02525	3.112509819	0	0	300	up
gene-PFY09_RS02530	2.856989066	0	0	300	up
gene-PFY09_RS03635	1.967905661	0	0	300	up
gene-PFY09_RS03680	1.822331551	0	0	300	up
gene-PFY09_RS03730	2.413631494	0	0	300	up
gene-PFY09_RS04465	3.156878201	0	0	300	up
gene-PFY09_RS04735	1.761303407	0	0	300	up
gene-PFY09_RS05125	2.441772838	0	0	300	up
gene-PFY09_RS05245	1.460481109	0	0	300	up
gene-PFY09_RS05250	2.363184665	0	0	300	up
gene-PFY09_RS06325	1.514887517	0	0	300	up
gene-PFY09_RS06755	2.00349483	0	0	300	up
gene-PFY09_RS07820	2.325316982	0	0	300	up
gene-PFY09_RS07830	3.610382337	0	0	300	up
gene-PFY09_RS08580	4.417458645	0	0	300	up
gene-PFY09_RS08740	3.109265669	0	0	300	up
gene-PFY09_RS08795	2.174635444	0	0	300	up
gene-PFY09_RS08980	2.767067612	0	0	300	up
gene-PFY09_RS08995	1.921067892	0	0	300	up

gene-PFY09_RS09000	2.414763058	0	0	300	up
gene-PFY09_RS09015	2.267336738	0	0	300	up
gene-PFY09_RS09195	2.793688412	0	0	300	up
gene-PFY09_RS09460	2.31652312	0	0	300	up
gene-PFY09_RS09775	5.188423895	0	0	300	up
gene-PFY09_RS10030	2.667275494	0	0	300	up
gene-PFY09_RS10155	2.678521534	0	0	300	up
gene-PFY09_RS10915	3.260531529	0	0	300	up
gene-PFY09_RS10925	4.057490174	0	0	300	up
gene-PFY09_RS11465	2.119650726	0	0	300	up
gene-PFY09_RS11535	2.118008371	0	0	300	up
gene-PFY09_RS11540	2.249491739	0	0	300	up
gene-PFY09_RS11620	2.900134274	0	0	300	up
gene-PFY09_RS11715	1.972106107	0	0	300	up
gene-PFY09_RS11755	2.921490618	0	0	300	up
gene-PFY09_RS11970	2.988741423	0	0	300	up
gene-PFY09_RS11995	2.441912857	0	0	300	up
gene-PFY09_RS12120	2.073829604	0	0	300	up
gene-PFY09_RS12125	2.679619361	0	0	300	up
gene-PFY09_RS12135	1.51660481	0	0	300	up
gene-PFY09_RS12715	2.048630453	0	0	300	up
gene-PFY09_RS12725	2.612970349	0	0	300	up

gene-PFY09_RS12790	3.474281555	0	0	300	up
gene-PFY09_RS12900	2.018270849	0	0	300	up
gene-PFY09_RS13980	3.284715876	0	0	300	up
gene-PFY09_RS14350	3.049641626	0	0	300	up
gene-PFY09_RS14840	5.610936226	0	0	300	up
gene-PFY09_RS15270	1.591985288	0	0	300	up
gene-PFY09_RS15700	2.07790404	0	0	300	up
gene-PFY09_RS15790	2.592495432	0	0	300	up
gene-PFY09_RS15800	2.179607908	0	0	300	up
gene-PFY09_RS15805	1.773293927	0	0	300	up
gene-PFY09_RS15820	3.003302009	0	0	300	up
gene-PFY09_RS16230	3.453960744	0	0	300	up
gene-PFY09_RS16235	3.005523213	0	0	300	up
gene-PFY09_RS17525	4.860564948	0	0	300	up
gene-PFY09_RS17635	2.65848208	0	0	300	up
gene-PFY09_RS17805	2.826579582	0	0	300	up
gene-PFY09_RS18575	3.617770535	0	0	300	up
gene-PFY09_RS18600	5.80239363	0	0	300	up
gene-PFY09_RS18605	3.006814479	0	0	300	up
gene-PFY09_RS18615	3.605918401	0	0	300	up
gene-PFY09_RS18635	6.385925968	0	0	300	up
gene-PFY09_RS18800	2.909378967	0	0	300	up

gene-PFY09_RS19410	3.083500511	0	0	300	up
gene-PFY09_RS19870	2.058709388	0	0	300	up
gene-PFY09_RS20005	2.343662361	0	0	300	up
gene-PFY09_RS20015	1.47933504	0	0	300	up
gene-PFY09_RS18350	4.071317302	3.8659E-289	1.2595E-287	286.8997927	up
gene-PFY09_RS18630	7.467612737	1.3895E-288	4.479E-287	286.3488177	up
gene-PFY09_RS01915	3.824888644	2.4547E-284	7.8292E-283	282.1062836	up
gene-PFY09_RS07825	2.358702455	3.2435E-282	1.0237E-280	279.9898119	up
gene-PFY09_RS10420	2.268822354	8.3399E-281	2.6051E-279	278.5841692	up
gene-PFY09_RS08330	2.221415916	1.5646E-276	4.8376E-275	274.3153702	up
gene-PFY09_RS09770	3.14004794	1.713E-274	5.243E-273	272.2804242	up
gene-PFY09_RS08525	1.714754662	2.7193E-274	8.2395E-273	272.0840979	up
gene-PFY09_RS11985	3.585966749	2.5186E-266	7.5557E-265	264.1217228	up
gene-PFY09_RS14200	2.937559798	3.81E-262	1.132E-260	259.9461454	up
gene-PFY09_RS18390	2.0502233	1.07E-260	3.1452E-259	258.5023539	up
gene-PFY09_RS05045	3.036657299	1.1679E-258	3.4026E-257	256.4681873	up
gene-PFY09_RS02160	1.516396167	1.1656E-255	3.3318E-254	253.4773249	up
gene-PFY09_RS19810	2.034261139	3.1297E-253	8.8626E-252	251.0524369	up
gene-PFY09_RS00590	1.662249263	3.4735E-250	9.7451E-249	248.011213	up
gene-PFY09_RS12130	1.476263667	1.085E-248	3.0161E-247	246.5205478	up
gene-PFY09_RS18175	1.6622747	5.0603E-248	1.3939E-246	245.8557735	up
gene-PFY09_RS03065	1.513443112	6.9651E-240	1.8676E-238	237.7287089	up

gene-PFY09_RS09250	1.271763977	1.4345E-238	3.8126E-237	236.4187753	up
gene-PFY09_RS08885	2.483813424	1.7197E-230	4.4919E-229	228.3475676	up
gene-PFY09_RS19735	2.381720399	5.2958E-228	1.3598E-226	225.8665105	up
gene-PFY09_RS02995	2.78769149	7.0715E-228	1.8006E-226	225.7445924	up
gene-PFY09_RS02715	1.540434033	7.4376E-217	1.8174E-215	214.7405454	up
gene-PFY09_RS04460	2.899315745	4.2212E-211	1.0151E-209	208.9934941	up
gene-PFY09_RS11815	1.940249524	9.1275E-211	2.1777E-209	208.6620098	up
gene-PFY09_RS10160	2.339087449	6.2426E-209	1.4663E-207	206.8337845	up
gene-PFY09_RS08920	1.921397027	2.0272E-205	4.7249E-204	203.3256087	up
gene-PFY09_RS20370	2.336833405	1.9614E-204	4.5366E-203	202.343268	up
gene-PFY09_RS09240	1.569452736	2.077E-200	4.7677E-199	198.3216947	up
gene-PFY09_RS01100	2.21285781	3.1665E-200	7.2139E-199	198.1418286	up
gene-PFY09_RS11700	1.924583812	1.1499E-197	2.5619E-196	195.5914436	up
gene-PFY09_RS19270	1.348005258	1.5791E-197	3.4924E-196	195.4568775	up
gene-PFY09_RS00660	1.834399392	4.4031E-196	9.6678E-195	194.0146743	up
gene-PFY09_RS15835	1.717640795	8.4815E-195	1.8488E-193	192.7331013	up
gene-PFY09_RS04160	1.547593868	1.4583E-194	3.1563E-193	192.5008244	up
gene-PFY09_RS18580	2.404319004	4.3201E-194	9.2836E-193	192.0322851	up
gene-PFY09_RS01805	2.10837697	1.4859E-192	3.1706E-191	190.4988612	up
gene-PFY09_RS02225	4.046599786	2.6982E-191	5.7171E-190	189.2428271	up
gene-PFY09_RS19275	2.179851651	2.2425E-189	4.7185E-188	187.3261917	up
gene-PFY09_RS09010	1.979025181	9.59E-188	1.9903E-186	185.7010903	up

gene-PFY09_RS09750	1.552345762	2.4025E-182	4.8856E-181	180.3110795	up
gene-PFY09_RS06135	1.660523111	8.5574E-177	1.7059E-175	174.7680566	up
gene-PFY09_RS07670	1.198200556	5.1226E-175	1.0145E-173	172.9937623	up
gene-PFY09_RS18400	1.224419799	2.3112E-170	4.5181E-169	168.3450434	up
gene-PFY09_RS18945	1.514625084	2.067E-169	4.0147E-168	167.3963445	up
gene-PFY09_RS16385	3.086732461	7.8338E-168	1.5023E-166	165.8232412	up
gene-PFY09_RS08790	1.512890738	1.0294E-165	1.9494E-164	163.7100918	up
gene-PFY09_RS16620	1.663064407	9.4905E-165	1.7861E-163	162.7480962	up
gene-PFY09_RS17620	1.42415066	3.1756E-162	5.9031E-161	160.2289191	up
gene-PFY09_RS01090	3.810567587	5.1662E-161	9.5449E-160	159.0202294	up
gene-PFY09_RS13620	1.743225387	1.5274E-159	2.8049E-158	157.552087	up
gene-PFY09_RS09455	2.521482873	1.3245E-157	2.3888E-156	155.6218255	up
gene-PFY09_RS02155	2.037662083	2.0365E-157	3.6513E-156	155.4375581	up
gene-PFY09_RS09760	1.645998062	1.2215E-154	2.1772E-153	152.6621074	up
gene-PFY09_RS08520	2.462659265	1.4435E-152	2.5577E-151	150.5921444	up
gene-PFY09_RS14215	2.502605224	8.5647E-152	1.5088E-150	149.821373	up
gene-PFY09_RS11920	1.295735516	1.3906E-151	2.4355E-150	149.6134164	up
gene-PFY09_RS04390	2.862416245	4.1725E-151	7.2658E-150	149.1387148	up
gene-PFY09_RS07450	1.332263916	4.2764E-149	7.4044E-148	147.1305127	up
gene-PFY09_RS05055	3.953485759	7.0775E-147	1.2116E-145	144.9166504	up
gene-PFY09_RS10270	3.192864033	1.2854E-146	2.188E-145	144.6599534	up
gene-PFY09_RS02235	3.355181614	4.7403E-144	8.024E-143	142.0956083	up

gene-PFY09_RS03875	1.670041207	1.0268E-143	1.7284E-142	141.7623589	up
gene-PFY09_RS15565	2.724350746	2.4679E-143	4.1313E-142	141.3839148	up
gene-PFY09_RS10935	4.359201278	9.3856E-143	1.5626E-141	140.8061648	up
gene-PFY09_RS15965	1.294596276	2.644E-140	4.3777E-139	138.3587517	up
gene-PFY09_RS01220	1.599808227	2.7413E-140	4.5142E-139	138.345415	up
gene-PFY09_RS16605	1.218012652	8.4497E-138	1.3618E-136	135.8658727	up
gene-PFY09_RS01395	2.047961149	5.1565E-137	8.2232E-136	135.0849594	up
gene-PFY09_RS03735	2.481568843	7.9046E-136	1.2474E-134	133.9039813	up
gene-PFY09_RS14190	1.424649198	1.1854E-132	1.8515E-131	130.7324852	up
gene-PFY09_RS00355	1.49724938	6.3923E-131	9.9326E-130	129.0029375	up
gene-PFY09_RS12035	1.797372353	5.4346E-130	8.4014E-129	128.0756483	up
gene-PFY09_RS04220	2.522424371	1.8628E-126	2.8222E-125	124.5494174	up
gene-PFY09_RS11975	3.670365261	4.7385E-126	7.1078E-125	124.1482635	up
gene-PFY09_RS18555	2.308508074	4.8571E-125	7.2498E-124	123.1396759	up
gene-PFY09_RS10095	1.297171759	4.22E-123	6.2679E-122	121.2028782	up
gene-PFY09_RS20380	1.202070184	1.9581E-122	2.8942E-121	120.5384761	up
gene-PFY09_RS11615	1.969911958	8.5565E-120	1.2525E-118	117.9022293	up
gene-PFY09_RS04530	1.735287993	1.6595E-118	2.3944E-117	116.6207952	up
gene-PFY09_RS00445	1.397817588	8.9963E-117	1.2919E-115	114.8887744	up
gene-PFY09_RS05050	3.465182578	3.2598E-116	4.6591E-115	114.3316962	up
gene-PFY09_RS16625	1.247417669	4.2071E-116	5.9847E-115	114.2229541	up
gene-PFY09_RS20490	1.827319567	2.8965E-115	4.0821E-114	113.3891196	up

gene-PFY09_RS02220	3.434914166	3.8135E-115	5.3495E-114	113.2716867	up
gene-PFY09_RS00565	2.348339464	1.7831E-114	2.4783E-113	112.6058502	up
gene-PFY09_RS16635	1.164940555	6.5091E-112	8.884E-111	110.0513921	up
gene-PFY09_RS19540	1.024144291	2.5058E-110	3.3896E-109	108.4698556	up
gene-PFY09_RS01365	2.275009915	7.0685E-110	9.4768E-109	108.0233385	up
gene-PFY09_RS15265	1.701481189	8.3485E-110	1.1144E-108	107.9529768	up
gene-PFY09_RS12075	1.032079563	3.2188E-109	4.2404E-108	107.3725934	up
gene-PFY09_RS08690	1.631281129	8.7941E-106	1.1485E-104	103.9398533	up
gene-PFY09_RS20495	1.183292974	1.5457E-104	1.9846E-103	102.7023373	up
gene-PFY09_RS01145	2.220514072	1.9076E-104	2.4286E-103	102.6146514	up
gene-PFY09_RS02130	1.055484005	2.6364E-104	3.3424E-103	102.4759393	up
gene-PFY09_RS00385	2.870241462	1.5822E-103	1.9892E-102	101.7013212	up
gene-PFY09_RS11760	1.224602888	7.2537E-103	9.0821E-102	101.0418153	up
gene-PFY09_RS06600	1.401128754	9.9823E-103	1.2396E-101	100.9067149	up
gene-PFY09_RS01350	1.207424195	1.9664E-102	2.4319E-101	100.6140523	up
gene-PFY09_RS16710	2.707880997	3.875E-102	4.77E-101	100.3212176	up
gene-PFY09_RS01155	2.354383982	4.9697E-101	5.9755E-100	99.22362726	up
gene-PFY09_RS09965	2.406623168	8.7046E-101	1.0425E-99	98.9819297	up
gene-PFY09_RS03320	1.209587743	8.2547E-100	9.7702E-99	98.01009697	up
gene-PFY09_RS09125	1.515247345	1.00965E-98	1.17663E-97	96.92936066	up
gene-PFY09_RS16615	1.216356921	1.67784E-96	1.94784E-95	94.71044717	up
gene-PFY09_RS04830	1.957384858	3.8849E-95	4.44198E-94	93.35242379	up

gene-PFY09_RS18045	1.174466855	4.9322E-94	5.59722E-93	92.25202788	up
gene-PFY09_RS16705	1.485134404	5.00162E-94	5.65481E-93	92.2475817	up
gene-PFY09_RS19405	2.154558293	5.13822E-94	5.78766E-93	92.23749731	up
gene-PFY09_RS07835	2.33731319	2.61489E-93	2.93449E-92	91.53246801	up
gene-PFY09_RS07595	2.001950672	9.18605E-93	1.02708E-91	90.98839764	up
gene-PFY09_RS03360	1.392940577	1.72971E-92	1.92685E-91	90.71515256	up
gene-PFY09_RS00570	2.151619753	6.06946E-89	6.59157E-88	87.18101119	up
gene-PFY09_RS20585	1.682203287	1.12427E-88	1.2123E-87	86.91639132	up
gene-PFY09_RS09220	1.083386057	4.79447E-88	5.1333E-87	86.28960298	up
gene-PFY09_RS13330	2.252218122	7.02381E-88	7.49371E-87	86.12530321	up
gene-PFY09_RS18570	1.044738437	1.74179E-87	1.8518E-86	85.73240594	up
gene-PFY09_RS18335	1.279273671	5.35495E-87	5.67325E-86	85.24616813	up
gene-PFY09_RS17585	1.590443303	2.14706E-86	2.26676E-85	84.64459476	up
gene-PFY09_RS11820	1.369546349	1.86695E-85	1.95739E-84	83.70832286	up
gene-PFY09_RS06760	1.659607573	8.67211E-85	9.02972E-84	83.04432578	up
gene-PFY09_RS15480	1.404876917	3.22696E-84	3.32574E-83	82.47811146	up
gene-PFY09_RS15560	1.416965836	2.23345E-83	2.27857E-82	81.64233825	up
gene-PFY09_RS08210	1.863416066	2.31981E-82	2.32749E-81	80.63311136	up
gene-PFY09_RS12205	1.410861952	3.94621E-82	3.92033E-81	80.40667697	up
gene-PFY09_RS05475	1.150789701	4.27432E-82	4.23241E-81	80.37341205	up
gene-PFY09_RS14240	1.239832829	1.0759E-81	1.05843E-80	79.97533636	up
gene-PFY09_RS01315	1.554297853	5.12784E-81	5.01205E-80	79.29998463	up

gene-PFY09_RS10920	3.153970917	5.40976E-81	5.27061E-80	79.27813946	up
gene-PFY09_RS06850	1.216420874	2.94859E-79	2.81837E-78	77.55000209	up
gene-PFY09_RS19865	1.750086554	3.27218E-79	3.11784E-78	77.50614672	up
gene-PFY09_RS07410	2.544835747	9.72142E-77	9.11947E-76	75.0400304	up
gene-PFY09_RS14770	1.221085246	2.65132E-76	2.47948E-75	74.6056395	up
gene-PFY09_RS08120	2.324458301	8.53378E-76	7.9317E-75	74.10063357	up
gene-PFY09_RS12820	2.294342707	2.36442E-75	2.19089E-74	73.65938	up
gene-PFY09_RS13145	1.211911856	6.67506E-74	6.11041E-73	72.21392989	up
gene-PFY09_RS19150	1.157193715	1.62199E-72	1.46705E-71	70.83355462	up
gene-PFY09_RS10630	1.10433647	2.62644E-72	2.36146E-71	70.62682001	up
gene-PFY09_RS15840	1.089122145	4.45341E-72	3.98048E-71	70.40006405	up
gene-PFY09_RS03020	1.970962872	1.77446E-71	1.56753E-70	69.80478543	up
gene-PFY09_RS16655	1.579913336	2.14318E-71	1.88774E-70	69.72405769	up
gene-PFY09_RS04155	1.53578098	3.22742E-71	2.82632E-70	69.54877834	up
gene-PFY09_RS18655	1.390121736	7.63183E-71	6.66411E-70	69.17625794	up
gene-PFY09_RS04405	2.818444	1.04031E-70	9.03189E-70	69.04422149	up
gene-PFY09_RS17710	1.816073436	3.59518E-70	3.1124E-69	68.50690507	up
gene-PFY09_RS20625	1.415811904	2.26455E-69	1.94379E-68	67.71135063	up
gene-PFY09_RS15070	2.091076337	7.54403E-68	6.43899E-67	66.19118255	up
gene-PFY09_RS07910	1.591159988	1.20637E-67	1.02389E-66	65.98974544	up
gene-PFY09_RS19945	1.882137925	1.67895E-67	1.42101E-66	65.84740184	up
gene-PFY09_RS07925	1.105837732	6.83857E-66	5.69255E-65	64.24469321	up

gene-PFY09_RS07900	1.585858571	9.40091E-66	7.80404E-65	64.10768037	up
gene-PFY09_RS13895	2.130180054	8.94212E-65	7.40291E-64	63.13059776	up
gene-PFY09_RS06435	1.016476923	4.23917E-64	3.49041E-63	62.45712391	up
gene-PFY09_RS14450	1.786332639	2.53358E-63	2.08042E-62	61.68184946	up
gene-PFY09_RS04990	2.009149838	1.28159E-61	1.0246E-60	59.98944601	up
gene-PFY09_RS03380	1.505357058	2.75726E-61	2.19855E-60	59.65786341	up
gene-PFY09_RS13410	1.760638988	3.36479E-61	2.67594E-60	59.57252407	up
gene-PFY09_RS19685	2.244589323	7.53564E-61	5.96162E-60	59.22463587	up
gene-PFY09_RS09330	1.188972992	1.26355E-60	9.9443E-60	59.00242571	up
gene-PFY09_RS17485	4.241453133	5.61556E-60	4.40807E-59	58.35575145	up
gene-PFY09_RS05380	1.141248032	7.43995E-60	5.82508E-59	58.23469807	up
gene-PFY09_RS18145	3.886025378	5.9225E-59	4.61315E-58	57.3360021	up
gene-PFY09_RS13890	1.90976186	1.14581E-58	8.85667E-58	57.05272934	up
gene-PFY09_RS18375	1.129867597	1.18476E-58	9.13442E-58	57.03931893	up
gene-PFY09_RS03715	1.442705968	2.23345E-58	1.7176E-57	56.76507788	up
gene-PFY09_RS03780	1.277892041	1.14818E-57	8.7853E-57	56.0562433	up
gene-PFY09_RS02360	1.833865848	6.69277E-57	5.08248E-56	55.29392468	up
gene-PFY09_RS10510	1.199017738	1.87176E-56	1.41786E-55	54.84836775	up
gene-PFY09_RS02655	1.308390851	2.51443E-56	1.8952E-55	54.72234415	up
gene-PFY09_RS07475	1.60173935	5.42557E-56	4.06918E-55	54.39049346	up
gene-PFY09_RS02245	3.176038405	1.31228E-55	9.79364E-55	54.00905587	up
gene-PFY09_RS08235	2.167251505	2.57829E-55	1.91946E-54	53.71682007	up

gene-PFY09_RS00270	1.77872749	5.30672E-55	3.90276E-54	53.40862828	up
gene-PFY09_RS12110	1.330224488	7.25068E-55	5.31951E-54	53.27412857	up
gene-PFY09_RS09320	1.57729848	8.04536E-55	5.88827E-54	53.23001241	up
gene-PFY09_RS16390	2.122151028	1.3504E-53	9.85953E-53	52.00614388	up
gene-PFY09_RS19415	1.448298787	6.77401E-53	4.91035E-52	51.30888783	up
gene-PFY09_RS15065	2.6249743	1.98417E-52	1.43144E-51	50.84422702	up
gene-PFY09_RS03515	3.459224074	3.12721E-52	2.24006E-51	50.6497408	up
gene-PFY09_RS14140	1.252848012	5.69381E-52	4.06893E-51	50.39052025	up
gene-PFY09_RS11695	1.498306917	6.54036E-52	4.66289E-51	50.33134495	up
gene-PFY09_RS13770	1.224449731	1.98727E-51	1.40688E-50	49.85174371	up
gene-PFY09_RS15555	1.302796712	4.15377E-51	2.93379E-50	49.5325717	up
gene-PFY09_RS01215	1.433817952	5.73596E-50	3.97711E-49	48.40043233	up
gene-PFY09_RS13370	1.76386493	7.0912E-50	4.90555E-49	48.30931186	up
gene-PFY09_RS06380	2.208679296	1.43961E-49	9.93623E-49	48.00277828	up
gene-PFY09_RS16275	1.048858532	1.48847E-49	1.02269E-48	47.99025688	up
gene-PFY09_RS02720	1.414565956	1.9939E-49	1.36686E-48	47.86427632	up
gene-PFY09_RS15755	1.061975083	4.32554E-49	2.95855E-48	47.52892124	up
gene-PFY09_RS06410	1.360551299	1.20247E-48	8.151E-48	47.08878913	up
gene-PFY09_RS10585	1.895904349	1.66697E-48	1.12744E-47	46.9479083	up
gene-PFY09_RS18610	2.22034581	2.3821E-48	1.60039E-47	46.79577319	up
gene-PFY09_RS13375	1.430324539	3.91855E-48	2.62102E-47	46.58153013	up
gene-PFY09_RS08175	1.646491055	8.39558E-48	5.60322E-47	46.25156258	up

gene-PFY09_RS16300	1.431084593	3.6993E-47	2.45271E-46	45.61035422	up
gene-PFY09_RS19290	1.615588235	8.13532E-47	5.37037E-46	45.26999568	up
gene-PFY09_RS10895	1.096688929	1.10247E-46	7.24618E-46	45.13989081	up
gene-PFY09_RS08685	1.833575871	2.42162E-46	1.58478E-45	44.80003158	up
gene-PFY09_RS18565	1.722018363	2.90925E-46	1.89979E-45	44.72129416	up
gene-PFY09_RS20465	1.484566339	4.99551E-46	3.25514E-45	44.48743024	up
gene-PFY09_RS05065	1.828995647	2.23882E-45	1.4464E-44	43.83971062	up
gene-PFY09_RS01810	2.185111886	2.84358E-45	1.82543E-44	43.73863365	up
gene-PFY09_RS16595	1.366764405	3.34701E-45	2.13954E-44	43.66967915	up
gene-PFY09_RS04470	1.698697427	4.64428E-45	2.96256E-44	43.52833256	up
gene-PFY09_RS08715	1.056053303	2.12695E-44	1.34264E-43	42.87204065	up
gene-PFY09_RS17640	2.801086709	7.54858E-43	4.69655E-42	41.32822095	up
gene-PFY09_RS11525	2.024039532	1.52488E-42	9.46801E-42	41.02374112	up
gene-PFY09_RS04150	1.278654262	2.30289E-42	1.42695E-41	40.84559214	up
gene-PFY09_RS04165	2.534350623	3.42053E-42	2.11515E-41	40.67465943	up
gene-PFY09_RS00310	1.082306398	4.28807E-42	2.63547E-41	40.57914237	up
gene-PFY09_RS05580	1.861482847	6.95397E-42	4.26529E-41	40.37005149	up
gene-PFY09_RS01085	3.853424391	1.00487E-41	6.15104E-41	40.21105119	up
gene-PFY09_RS04275	1.245613199	3.07672E-41	1.87198E-40	39.72769867	up
gene-PFY09_RS19620	1.742105758	3.28397E-41	1.99407E-40	39.70025934	up
gene-PFY09_RS11980	2.966955156	2.31062E-40	1.37818E-39	38.86069266	up
gene-PFY09_RS12250	1.369961168	5.39308E-40	3.20412E-39	38.49429072	up

gene-PFY09_RS07895	1.389290577	1.841E-39	1.08738E-38	37.96362038	up
gene-PFY09_RS08785	2.768173078	2.40111E-39	1.41269E-38	37.84995243	up
gene-PFY09_RS20615	1.262534572	2.75156E-39	1.61574E-38	37.7916284	up
gene-PFY09_RS16540	1.46125458	4.67535E-39	2.73481E-38	37.56307257	up
gene-PFY09_RS05075	1.409776025	5.69767E-39	3.32638E-38	37.47802753	up
gene-PFY09_RS02705	1.54480485	7.1033E-39	4.1311E-38	37.38393469	up
gene-PFY09_RS05215	1.895866613	1.29035E-38	7.44714E-38	37.12801022	up
gene-PFY09_RS10620	1.018854208	8.02845E-38	4.5812E-37	36.33902031	up
gene-PFY09_RS08985	1.472235658	9.7072E-38	5.51835E-37	36.25819065	up
gene-PFY09_RS00200	1.043685778	1.83141E-37	1.03529E-36	35.98493706	up
gene-PFY09_RS17955	1.712910745	1.9949E-37	1.12561E-36	35.94861079	up
gene-PFY09_RS08575	2.656901307	4.71482E-37	2.65045E-36	35.57668089	up
gene-PFY09_RS15080	1.216651536	8.3753E-37	4.69079E-36	35.3287541	up
gene-PFY09_RS08585	1.280625191	3.89185E-36	2.16373E-35	34.66479773	up
gene-PFY09_RS05205	1.1436135	4.11727E-36	2.28486E-35	34.6411409	up
gene-PFY09_RS18240	1.37709102	6.17895E-36	3.41024E-35	34.46721469	up
gene-PFY09_RS18620	2.595410021	6.35399E-36	3.50047E-35	34.4558736	up
gene-PFY09_RS10985	2.929735626	7.00376E-36	3.85143E-35	34.41437744	up
gene-PFY09_RS05225	1.850970932	2.15785E-35	1.1802E-34	33.92804593	up
gene-PFY09_RS01985	1.038566269	2.76807E-35	1.51121E-34	33.82067406	up
gene-PFY09_RS01070	3.068386159	3.96409E-34	2.13343E-33	32.67092175	up
gene-PFY09_RS13975	1.029505894	4.35069E-34	2.3332E-33	32.63204763	up

gene-PFY09_RS10045	1.178300146	6.0843E-34	3.2514E-33	32.48792991	up
gene-PFY09_RS09705	2.188447805	7.45431E-34	3.97402E-33	32.40076975	up
gene-PFY09_RS06285	1.534863091	7.46277E-34	3.97402E-33	32.40076975	up
gene-PFY09_RS16255	1.001885809	1.40929E-33	7.46529E-33	32.12695331	up
gene-PFY09_RS14160	1.294411894	1.80538E-32	9.44785E-32	31.02466685	up
gene-PFY09_RS00440	1.402774198	1.90481E-32	9.95101E-32	31.00213287	up
gene-PFY09_RS14070	2.450882422	6.6031E-32	3.41423E-31	30.46670742	up
gene-PFY09_RS19750	1.180667134	8.40837E-32	4.33288E-31	30.36322314	up
gene-PFY09_RS17465	2.512440817	1.77954E-31	9.09277E-31	30.04130372	up
gene-PFY09_RS03610	1.111840347	3.54578E-31	1.79661E-30	29.74554736	up
gene-PFY09_RS13365	1.258718015	6.33807E-31	3.1954E-30	29.49547485	up
gene-PFY09_RS03525	1.378371351	1.1411E-30	5.72438E-30	29.24227125	up
gene-PFY09_RS01065	3.66559232	4.86581E-30	2.413E-29	28.61744348	up
gene-PFY09_RS18950	1.284107137	7.53862E-30	3.7202E-29	28.42943402	up
gene-PFY09_RS08020	1.294168848	8.6843E-30	4.27166E-29	28.36940332	up
gene-PFY09_RS04280	1.840365377	1.29458E-29	6.34721E-29	28.197417	up
gene-PFY09_RS04835	1.84822768	3.0751E-29	1.49081E-28	27.82657848	up
gene-PFY09_RS13550	1.33771877	3.70395E-29	1.79281E-28	27.74646692	up
gene-PFY09_RS16015	1.08173022	4.3911E-29	2.11864E-28	27.67394324	up
gene-PFY09_RS13600	1.751929945	2.22104E-28	1.05482E-27	26.97682192	up
gene-PFY09_RS15830	2.230073389	4.73115E-28	2.22599E-27	26.65247647	up
gene-PFY09_RS03640	1.232390035	4.74986E-28	2.23133E-27	26.65143646	up

gene-PFY09_RS10930	3.179543478	9.62799E-28	4.48812E-27	26.34793507	up
gene-PFY09_RS12610	1.419145984	6.45287E-27	2.96695E-26	25.52769012	up
gene-PFY09_RS01080	3.582460819	1.01754E-26	4.6573E-26	25.33186557	up
gene-PFY09_RS03700	1.244429047	1.09323E-26	4.9812E-26	25.30266561	up
gene-PFY09_RS02185	1.004062663	1.95268E-26	8.85723E-26	25.05270187	up
gene-PFY09_RS18625	1.81652432	6.53212E-26	2.9322E-25	24.53280709	up
gene-PFY09_RS02550	1.23703473	6.55202E-26	2.93678E-25	24.53212853	up
gene-PFY09_RS05505	2.498763019	1.70639E-25	7.6259E-25	24.11770894	up
gene-PFY09_RS04385	1.308365874	1.75191E-25	7.81781E-25	24.10691482	up
gene-PFY09_RS01075	2.876104638	2.59027E-25	1.14912E-24	23.93963301	up
gene-PFY09_RS07480	1.829667209	3.33197E-25	1.476E-24	23.83091225	up
gene-PFY09_RS14030	2.083182874	3.44023E-25	1.52174E-24	23.81766032	up
gene-PFY09_RS05035	1.374513257	3.9482E-25	1.74135E-24	23.75911461	up
gene-PFY09_RS04310	1.115205482	4.18727E-25	1.84143E-24	23.73484587	up
gene-PFY09_RS08860	1.72526843	7.47203E-25	3.26229E-24	23.48647801	up
gene-PFY09_RS04870	2.395623932	1.94394E-24	8.42652E-24	23.07435159	up
gene-PFY09_RS01555	1.74127847	3.57196E-24	1.54394E-23	22.81136933	up
gene-PFY09_RS14885	2.375808203	7.0162E-24	3.0112E-23	22.52126003	up
gene-PFY09_RS08590	1.987441035	2.90211E-23	1.22813E-22	21.91075634	up
gene-PFY09_RS14290	1.083150862	6.8144E-23	2.86375E-22	21.5430653	up
gene-PFY09_RS18295	1.866146094	9.51008E-23	3.99107E-22	21.39891053	up
gene-PFY09_RS20235	2.305584591	1.51655E-22	6.32069E-22	21.1992355	up

gene-PFY09_RS04370	1.240072806	2.02719E-22	8.36836E-22	21.07735981	up
gene-PFY09_RS09765	1.809820303	2.7123E-22	1.11661E-21	20.95209784	up
gene-PFY09_RS19950	1.003733028	4.2583E-22	1.74596E-21	20.75796585	up
gene-PFY09_RS05570	1.679174212	5.48907E-22	2.24755E-21	20.64828994	up
gene-PFY09_RS12465	1.070023503	7.70162E-22	3.13655E-21	20.5035484	up
gene-PFY09_RS17970	1.264160995	9.01795E-22	3.66279E-21	20.43618841	up
gene-PFY09_RS02110	2.646656425	2.53106E-21	1.01309E-20	19.99435091	up
gene-PFY09_RS10050	1.370600704	3.74786E-21	1.49225E-20	19.82615812	up
gene-PFY09_RS05130	1.693152592	1.45424E-20	5.72254E-20	19.24241152	up
gene-PFY09_RS05060	1.55210994	1.49986E-20	5.89438E-20	19.22956201	up
gene-PFY09_RS02230	2.727633545	1.55557E-20	6.08964E-20	19.21540841	up
gene-PFY09_RS11890	1.795078747	8.39592E-20	3.22429E-19	18.4915661	up
gene-PFY09_RS18085	1.406074461	1.63443E-19	6.22151E-19	18.20610451	up
gene-PFY09_RS08865	2.043097807	2.65544E-19	1.00575E-18	17.9975106	up
gene-PFY09_RS12980	1.059786737	6.06593E-19	2.26911E-18	17.64414472	up
gene-PFY09_RS18355	2.458568872	6.57637E-19	2.45702E-18	17.60959207	up
gene-PFY09_RS06315	1.072800896	9.03712E-19	3.3557E-18	17.4742175	up
gene-PFY09_RS02250	2.869837201	1.78648E-18	6.56125E-18	17.18301368	up
gene-PFY09_RS19820	1.550524034	2.93195E-18	1.07163E-17	16.96995559	up
gene-PFY09_RS13320	1.09834063	2.99759E-18	1.0943E-17	16.96086333	up
gene-PFY09_RS13380	1.302389051	8.52536E-18	3.07522E-17	16.51212383	up
gene-PFY09_RS01425	1.506823767	1.41343E-17	5.05631E-17	16.29616667	up

gene-PFY09_RS05020	1.556891982	8.45624E-17	2.97244E-16	15.52688755	up
gene-PFY09_RS03530	1.024929329	8.63254E-17	3.03089E-16	15.51842954	up
gene-PFY09_RS13395	1.202731267	1.19604E-16	4.18477E-16	15.37832867	up
gene-PFY09_RS08800	1.549489585	1.28964E-16	4.50703E-16	15.34610911	up
gene-PFY09_RS06685	1.307636699	1.32677E-16	4.62083E-16	15.33528016	up
gene-PFY09_RS15920	1.298511842	2.16221E-16	7.47887E-16	15.12616401	up
gene-PFY09_RS08780	1.119540818	4.46296E-16	1.52972E-15	14.81538709	up
gene-PFY09_RS15615	1.056627492	4.646E-16	1.59066E-15	14.7984215	up
gene-PFY09_RS17715	1.396694574	8.30449E-16	2.82154E-15	14.54951424	up
gene-PFY09_RS16550	1.244383419	1.21224E-15	4.08576E-15	14.38872731	up
gene-PFY09_RS11740	1.459341402	4.27177E-15	1.4115E-14	13.85031896	up
gene-PFY09_RS11475	1.396294269	5.22549E-15	1.72288E-14	13.7637457	up
gene-PFY09_RS19880	2.544062888	9.17904E-15	3.01001E-14	13.52143222	up
gene-PFY09_RS12965	1.223946032	1.86303E-14	6.05035E-14	13.21821922	up
gene-PFY09_RS08810	2.753312355	2.59852E-14	8.39393E-14	13.07603456	up
gene-PFY09_RS03240	2.027294677	3.84958E-14	1.23562E-13	12.90811647	up
gene-PFY09_RS17470	3.050743973	5.0692E-14	1.62193E-13	12.78996779	up
gene-PFY09_RS10055	1.124422158	5.67375E-14	1.81345E-13	12.74149534	up
gene-PFY09_RS14865	1.025401937	1.75405E-13	5.50183E-13	12.25949304	up
gene-PFY09_RS18940	1.108294725	4.16651E-13	1.2935E-12	11.88823445	up
gene-PFY09_RS14845	1.162944571	5.31412E-13	1.6464E-12	11.78346455	up
gene-PFY09_RS03725	1.418923603	5.85326E-13	1.80789E-12	11.74282833	up

gene-PFY09_RS15400	1.772396352	7.445E-13	2.29252E-12	11.63968776	up
gene-PFY09_RS01110	1.491297473	8.26486E-13	2.54239E-12	11.59475808	up
gene-PFY09_RS19285	1.857483133	9.97342E-13	3.06486E-12	11.51358999	up
gene-PFY09_RS04375	1.711020865	1.06753E-12	3.2739E-12	11.48493454	up
gene-PFY09_RS01105	1.68601417	2.23383E-12	6.80252E-12	11.16733027	up
gene-PFY09_RS13405	1.20952793	2.50805E-12	7.59938E-12	11.11922179	up
gene-PFY09_RS09065	2.824236394	3.24089E-12	9.78078E-12	11.00962658	up
gene-PFY09_RS10235	2.021372833	5.48278E-12	1.63673E-11	10.78602263	up
gene-PFY09_RS05070	1.87720103	6.12061E-12	1.82355E-11	10.73908345	up
gene-PFY09_RS14230	1.896301778	6.6492E-12	1.9752E-11	10.70438809	up
gene-PFY09_RS05825	1.138337685	7.14334E-12	2.11784E-11	10.67410721	up
gene-PFY09_RS06235	2.475778913	9.10874E-12	2.67956E-11	10.5719365	up
gene-PFY09_RS19860	1.497721185	1.04509E-11	3.07141E-11	10.51266231	up
gene-PFY09_RS03750	1.351631144	1.56758E-11	4.57149E-11	10.33994219	up
gene-PFY09_RS05545	1.890563951	1.63352E-11	4.7455E-11	10.32371771	up
gene-PFY09_RS13725	1.338749902	1.8414E-11	5.3443E-11	10.27210943	up
gene-PFY09_RS13000	1.079548903	2.1837E-11	6.31958E-11	10.19931153	up
gene-PFY09_RS20470	1.5570561	8.85396E-11	2.49326E-10	9.603232144	up
gene-PFY09_RS15545	1.185508408	1.34969E-10	3.77613E-10	9.422953295	up
gene-PFY09_RS16470	1.069458523	1.42231E-10	3.97197E-10	9.400993774	up
gene-PFY09_RS15675	1.842798901	1.73884E-10	4.82038E-10	9.316918366	up
gene-PFY09_RS18500	3.786917601	2.14337E-10	5.93639E-10	9.226477543	up

gene-PFY09_RS20240	1.395735984	2.66512E-10	7.34787E-10	9.133838673	up
gene-PFY09_RS01095	1.856839079	3.11886E-10	8.54444E-10	9.068316326	up
gene-PFY09_RS00575	1.352913693	7.32353E-10	1.97598E-09	8.704216783	up
gene-PFY09_RS17530	1.097262689	1.01907E-09	2.70857E-09	8.567260131	up
gene-PFY09_RS12915	1.024837536	1.14973E-09	3.03986E-09	8.517145866	up
gene-PFY09_RS10240	1.285299771	1.1714E-09	3.09445E-09	8.509416781	up
gene-PFY09_RS14335	1.183548464	1.6636E-09	4.36802E-09	8.35971538	up
gene-PFY09_RS05345	1.680888762	1.68993E-09	4.43333E-09	8.353270279	up
gene-PFY09_RS13810	2.058017846	2.05493E-09	5.35837E-09	8.270967316	up
gene-PFY09_RS14870	1.268596238	2.25509E-09	5.84509E-09	8.233208753	up
gene-PFY09_RS20230	1.394675283	2.39781E-09	6.20441E-09	8.207299222	up
gene-PFY09_RS04620	1.300319439	2.97984E-09	7.68418E-09	8.114402363	up
gene-PFY09_RS11750	1.105976211	3.70332E-09	9.48526E-09	8.022950917	up
gene-PFY09_RS05040	1.657731511	4.41509E-09	1.12512E-08	7.948799917	up
gene-PFY09_RS02255	2.592518058	4.69603E-09	1.19471E-08	7.92273855	up
gene-PFY09_RS03370	1.274067606	5.30161E-09	1.34538E-08	7.871154255	up
gene-PFY09_RS05110	1.373342352	6.41436E-09	1.62233E-08	7.789860643	up
gene-PFY09_RS08115	2.603292733	7.36907E-09	1.86069E-08	7.730326032	up
gene-PFY09_RS16680	1.396469769	7.5752E-09	1.91115E-08	7.71870611	up
gene-PFY09_RS05550	1.165003265	8.22605E-09	2.07362E-08	7.683270302	up
gene-PFY09_RS00395	1.583435808	1.18741E-08	2.96851E-08	7.527460961	up
gene-PFY09_RS18550	1.537357167	1.47666E-08	3.6795E-08	7.434211635	up

gene-PFY09_RS16405	1.263883074	1.95849E-08	4.86411E-08	7.31299634	up
gene-PFY09_RS04500	1.256414462	2.0301E-08	5.03372E-08	7.298111109	up
gene-PFY09_RS02240	3.862012359	2.32495E-08	5.7554E-08	7.239924375	up
gene-PFY09_RS12445	1.367777864	2.43627E-08	6.02112E-08	7.220322686	up
gene-PFY09_RS10590	1.344652337	2.81101E-08	6.92469E-08	7.159599731	up
gene-PFY09_RS12210	1.432200968	2.84075E-08	6.99227E-08	7.155381677	up
gene-PFY09_RS13155	1.659327161	4.97036E-08	1.21355E-07	6.915941687	up
gene-PFY09_RS18385	1.054056419	5.39968E-08	1.3152E-07	6.881009562	up
gene-PFY09_RS14155	1.088785732	6.65689E-08	1.61492E-07	6.791848341	up
gene-PFY09_RS01115	1.252793826	1.02885E-07	2.47021E-07	6.607265621	up
gene-PFY09_RS00650	1.010757557	1.21873E-07	2.91917E-07	6.534740217	up
gene-PFY09_RS19075	1.049117183	2.54331E-07	5.98776E-07	6.222735802	up
gene-PFY09_RS09175	1.026757676	3.40439E-07	7.9532E-07	6.099458346	up
gene-PFY09_RS13755	1.036525151	4.01557E-07	9.34498E-07	6.0294215	up
gene-PFY09_RS06810	1.168148992	4.43391E-07	1.02869E-06	5.987713724	up
gene-PFY09_RS07905	1.667081199	4.50316E-07	1.04396E-06	5.981315197	up
gene-PFY09_RS03050	1.212623542	4.65E-07	1.07533E-06	5.968459497	up
gene-PFY09_RS05305	1.116736814	5.66E-07	1.30219E-06	5.88532609	up
gene-PFY09_RS12480	1.323153896	6.50737E-07	1.49035E-06	5.826711843	up
gene-PFY09_RS10575	1.728857967	1.34242E-06	3.03094E-06	5.518422703	up
gene-PFY09_RS12365	1.32490341	1.56099E-06	3.51397E-06	5.454201808	up
gene-PFY09_RS08165	2.873361229	2.7291E-06	6.06243E-06	5.217353025	up

gene-PFY09_RS19835	1.308047939	4.37701E-06	9.59648E-06	5.017888215	up
gene-PFY09_RS00220	1.151710685	4.57897E-06	1.00248E-05	4.998925838	up
gene-PFY09_RS04170	1.901721021	4.80279E-06	1.0492E-05	4.979140471	up
gene-PFY09_RS05255	1.647241746	1.05721E-05	2.26224E-05	4.645461327	up
gene-PFY09_RS02335	1.658519084	1.13453E-05	2.42427E-05	4.615418871	up
gene-PFY09_RS19280	1.151736488	1.25275E-05	2.66748E-05	4.573899156	up
gene-PFY09_RS18125	1.276103976	1.26715E-05	2.69625E-05	4.56923952	up
gene-PFY09_RS09500	2.218902848	1.39616E-05	2.96245E-05	4.52834892	up
gene-PFY09_RS05560	2.431592929	1.66178E-05	3.50885E-05	4.454835522	up
gene-PFY09_RS07080	1.304168499	1.74997E-05	3.68735E-05	4.433285654	up
gene-PFY09_RS02265	1.532629184	2.30428E-05	4.82181E-05	4.316790304	up
gene-PFY09_RS18365	1.221037412	2.91343E-05	6.0755E-05	4.216417978	up
gene-PFY09_RS01120	1.777911507	3.49483E-05	7.248E-05	4.139781586	up
gene-PFY09_RS20225	2.211729875	3.66984E-05	7.59538E-05	4.119450742	up
gene-PFY09_RS18360	1.077190539	4.54397E-05	9.35978E-05	4.028734379	up
gene-PFY09_RS13605	2.927198386	7.46314E-05	0.000150856	3.821437195	up
gene-PFY09_RS15875	1.083436714	0.000110587	0.000220592	3.656410828	up
gene-PFY09_RS00275	1.018184291	0.000112149	0.000223414	3.650890175	up
gene-PFY09_RS12825	2.084495404	0.000195972	0.000383095	3.416693972	up
gene-PFY09_RS13045	1.22650586	0.000207756	0.000405083	3.392455624	up
gene-PFY09_RS04175	1.402050767	0.000214204	0.000416584	3.3802974	up
gene-PFY09_RS03300	1.73254846	0.000279531	0.000541201	3.266641485	up

gene-PFY09_RS17475	3.393482826	0.000326259	0.000625675	3.203651374	up
gene-PFY09_RS09165	1.371768947	0.000361744	0.000691536	3.160185263	up
gene-PFY09_RS17645	1.445934743	0.000409016	0.000776029	3.110121854	up
gene-PFY09_RS01275	1.204904249	0.000617981	0.001153716	2.937900969	up
gene-PFY09_RS13110	1.260296136	0.000663509	0.001235669	2.908098008	up
gene-PFY09_RS13070	1.274707535	0.001032471	0.001901756	2.720845225	up
gene-PFY09_RS11610	1.6397477	0.00139221	0.002538144	2.595483685	up
gene-PFY09_RS14055	1.002888908	0.001398317	0.002547746	2.593843832	up
gene-PFY09_RS13695	1.318684048	0.001888115	0.003401301	2.468354875	up
gene-PFY09_RS00540	1.600662591	0.002018335	0.003626224	2.440545411	up
gene-PFY09_RS05645	1.023882151	0.002018957	0.003626224	2.440545411	up
gene-PFY09_RS14205	1.025485194	0.002306627	0.004121099	2.384986992	up
gene-PFY09_RS04180	1.119676254	0.002640159	0.004683654	2.32941516	up
gene-PFY09_RS14065	2.324863047	0.00269894	0.004781853	2.320403741	up
gene-PFY09_RS16055	1.33860564	0.003124514	0.005497838	2.259808045	up
gene-PFY09_RS00615	1.230577918	0.003138467	0.005519185	2.258125059	up
gene-PFY09_RS05180	1.450423197	0.003354441	0.005868335	2.231485131	up
gene-PFY09_RS01455	2.040878582	0.00386664	0.006710149	2.173267864	up
gene-PFY09_RS01900	1.98581442	0.0038716	0.00671491	2.1729598	up
gene-PFY09_RS05585	1.014792387	0.003902432	0.006764513	2.169763479	up
gene-PFY09_RS10335	1.156157066	0.00435559	0.007524195	2.123539971	up
gene-PFY09_RS15060	1.429047627	0.004423921	0.00763788	2.117027162	up

gene-PFY09_RS04190	1.647280024	0.004760125	0.008171772	2.087683734	up
gene-PFY09_RS11585	1.263572601	0.005168314	0.00882253	2.054406856	up
gene-PFY09_RS00520	1.305646635	0.011107941	0.018301827	1.737505545	up
gene-PFY09_RS17755	1.504924226	0.011225825	0.018486005	1.733156924	up
gene-PFY09_RS18595	1.315625883	0.013746745	0.022442153	1.64893548	up
gene-PFY09_RS03025	1.013088106	0.0138411	0.022584024	1.64619868	up
gene-PFY09_RS14990	1.451314414	0.014153386	0.023056322	1.637209965	up
gene-PFY09_RS14090	1.185301735	0.01507859	0.024471412	1.611340963	up
gene-PFY09_RS11575	2.437454534	0.018084752	0.028993015	1.537706617	up
gene-PFY09_RS13610	1.004233176	0.018311694	0.029310318	1.532979472	up
gene-PFY09_RS11885	1.20656113	0.019235048	0.030642584	1.51367462	up
gene-PFY09_RS18830	1.040291716	0.021088318	0.033436736	1.475776126	up
gene-PFY09_RS16375	1.095661736	0.023691267	0.037310051	1.428174155	up
gene-PFY09_RS03520	2.649639272	0.026502963	0.041457914	1.382392558	up
gene-PFY09_RS08870	1.318679738	0.028331465	0.044045325	1.356100178	up
gene-PFY09_RS02565	2.086579417	0.028472477	0.044241848	1.354166737	up
gene-PFY09_RS17240	1.122287823	0.035688251	0.054669061	1.262258389	up
gene-PFY09_RS03090	1.19984075	0.035832865	0.05486285	1.260721637	up
gene-PFY09_RS17500	2.52098773	0.043748512	0.066080754	1.179925013	up
gene-PFY09_RS07660	1.252448015	0.04480915	0.067581745	1.170170601	up
gene-PFY09_RS03265	-2.123432699	0	0	300	down
gene-PFY09_RS03285	-2.39991687	0	0	300	down

gene-PFY09_RS04075	-2.17660694	0	0	300	down
gene-PFY09_RS06240	-1.99711674	0	0	300	down
gene-PFY09_RS06245	-2.100524123	0	0	300	down
gene-PFY09_RS06735	-2.916438117	0	0	300	down
gene-PFY09_RS06920	-2.694900582	0	0	300	down
gene-PFY09_RS07005	-2.289921107	0	0	300	down
gene-PFY09_RS08360	-1.83503983	0	0	300	down
gene-PFY09_RS08895	-3.196869432	0	0	300	down
gene-PFY09_RS09835	-1.274025699	0	0	300	down
gene-PFY09_RS09905	-2.543648604	0	0	300	down
gene-PFY09_RS09915	-2.956457751	0	0	300	down
gene-PFY09_RS11070	-1.589392978	0	0	300	down
gene-PFY09_RS12000	-1.826744172	0	0	300	down
gene-PFY09_RS15090	-1.846664963	0	0	300	down
gene-PFY09_RS15165	-1.859097894	0	0	300	down
gene-PFY09_RS17925	-6.044657803	0	0	300	down
gene-PFY09_RS17930	-4.819005752	0	0	300	down
gene-PFY09_RS18255	-2.247106696	0	0	300	down
gene-PFY09_RS19040	-1.990975467	0	0	300	down
gene-PFY09_RS19045	-2.007725645	0	0	300	down
gene-PFY09_RS19375	-2.263505588	0	0	300	down
gene-PFY09_RS15095	-1.503505436	6.5981E-256	1.904E-254	253.7203282	down

gene-PFY09_RS03915	-1.742533762	1.2182E-246	3.3254E-245	244.4781501	down
gene-PFY09_RS12095	-2.210847298	1.2047E-244	3.2592E-243	242.4868901	down
gene-PFY09_RS19050	-1.211668304	9.9536E-237	2.6226E-235	234.5812739	down
gene-PFY09_RS02390	-1.627874145	1.9757E-228	5.1166E-227	226.2910161	down
gene-PFY09_RS11080	-2.080529157	1.3299E-224	3.358E-223	222.4739169	down
gene-PFY09_RS09910	-2.544581395	3.554E-223	8.8996E-222	221.0506276	down
gene-PFY09_RS15020	-1.75091374	3.9135E-221	9.7197E-220	219.0123492	down
gene-PFY09_RS11075	-2.838000032	1.8319E-218	4.5127E-217	216.3455626	down
gene-PFY09_RS14345	-2.115088325	1.4444E-211	3.5012E-210	209.4557784	down
gene-PFY09_RS06925	-1.148541228	4.5759E-210	1.0832E-208	207.9652932	down
gene-PFY09_RS10455	-1.082173682	1.1386E-199	2.5746E-198	197.5892972	down
gene-PFY09_RS00390	-1.475438151	5.318E-199	1.1936E-197	196.9231461	down
gene-PFY09_RS02730	-2.113797521	3.3821E-189	7.0674E-188	187.1507381	down
gene-PFY09_RS12005	-1.754492602	2.5002E-187	5.1535E-186	185.2878983	down
gene-PFY09_RS09860	-1.107269821	2.7447E-185	5.6192E-184	183.2503245	down
gene-PFY09_RS09100	-2.224262781	1.3253E-181	2.6772E-180	179.5723185	down
gene-PFY09_RS11455	-2.06861611	3.8938E-180	7.8134E-179	178.1071613	down
gene-PFY09_RS03570	-2.76052542	1.2956E-172	2.5491E-171	170.5936198	down
gene-PFY09_RS19370	-1.303335381	6.9544E-168	1.3422E-166	165.8721976	down
gene-PFY09_RS08900	-5.021475113	2.502E-166	4.768E-165	164.3216602	down
gene-PFY09_RS10025	-1.488572299	4.3329E-163	8.1042E-162	161.0912915	down
gene-PFY09_RS17935	-3.96357009	1.8991E-158	3.4664E-157	156.4601179	down

gene-PFY09_RS02395	-1.108103978	4.5843E-158	8.3177E-157	156.0799976	down
gene-PFY09_RS04315	-1.588762743	5.3212E-149	9.161E-148	147.038059	down
gene-PFY09_RS06230	-1.569453426	3.5787E-140	5.8614E-139	138.232002	down
gene-PFY09_RS15170	-2.004170934	1.7295E-138	2.8023E-137	136.5524849	down
gene-PFY09_RS08250	-1.129714218	3.7435E-137	6.0014E-136	135.2217451	down
gene-PFY09_RS09115	-1.688905541	1.7205E-136	2.7293E-135	134.5639411	down
gene-PFY09_RS09390	-2.311320965	5.4741E-135	8.594E-134	133.0658027	down
gene-PFY09_RS05395	-1.567909862	4.1168E-126	6.2059E-125	124.207192	down
gene-PFY09_RS11710	-1.031022105	2.6162E-121	3.848E-120	119.4147616	down
gene-PFY09_RS10265	-1.194395138	6.0039E-116	8.5009E-115	114.0705357	down
gene-PFY09_RS07735	-2.292870574	1.3033E-114	1.8198E-113	112.7399672	down
gene-PFY09_RS06425	-1.166522777	2.0159E-112	2.7765E-111	110.5565062	down
gene-PFY09_RS09790	-1.753561376	3.8285E-112	5.249E-111	110.2799249	down
gene-PFY09_RS02820	-1.201040459	9.9173E-110	1.318E-108	107.8800987	down
gene-PFY09_RS19995	-1.226124338	2.4162E-109	3.1969E-108	107.4952652	down
gene-PFY09_RS08380	-1.727898318	1.519E-108	1.9924E-107	106.7006224	down
gene-PFY09_RS06575	-1.579210933	7.3103E-105	9.5065E-104	103.0219796	down
gene-PFY09_RS06730	-1.745645161	1.5211E-104	1.9613E-103	102.7074617	down
gene-PFY09_RS01945	-1.973788931	9.914E-103	1.2362E-101	100.9079159	down
gene-PFY09_RS06915	-1.424345474	4.4967E-102	5.4939E-101	100.2601195	down
gene-PFY09_RS15490	-3.739745022	1.1654E-101	1.4181E-100	99.84829359	down
gene-PFY09_RS04420	-1.699967198	3.6926E-101	4.4575E-100	99.35090456	down

gene-PFY09_RS14360	-1.160148334	1.2944E-100	1.5441E-99	98.81133284	down
gene-PFY09_RS12775	-3.004501767	1.7826E-100	2.1182E-99	98.67403563	down
gene-PFY09_RS01420	-1.168752178	3.1471E-99	3.69596E-98	97.43227303	down
gene-PFY09_RS15120	-1.103573323	5.55882E-96	6.42871E-95	94.19187602	down
gene-PFY09_RS20430	-2.157433171	8.55065E-96	9.85113E-95	94.00651402	down
gene-PFY09_RS20755	-2.336536595	1.1158E-95	1.28064E-94	93.89257372	down
gene-PFY09_RS12745	-2.958185963	6.09525E-95	6.94308E-94	93.15844759	down
gene-PFY09_RS15205	-1.149835677	6.72255E-91	7.43406E-90	89.12877403	down
gene-PFY09_RS02300	-1.111973265	3.14371E-90	3.45125E-89	88.46202348	down
gene-PFY09_RS14495	-1.907870007	1.92445E-88	2.06776E-87	86.68449914	down
gene-PFY09_RS20410	-1.324584519	4.91149E-86	5.16729E-85	84.28673689	down
gene-PFY09_RS15145	-1.55656619	2.79077E-85	2.91587E-84	83.5352318	down
gene-PFY09_RS07000	-1.410340104	8.28809E-84	8.4841E-83	82.07139436	down
gene-PFY09_RS15110	-2.094011526	2.8919E-83	2.94042E-82	81.5315904	down
gene-PFY09_RS02050	-1.038986677	6.75404E-83	6.8444E-82	81.16466461	down
gene-PFY09_RS10685	-1.398056649	1.00709E-82	1.01716E-81	80.99261231	down
gene-PFY09_RS18030	-1.124283499	2.13824E-82	2.15244E-81	80.66706807	down
gene-PFY09_RS06265	-2.271074916	2.35179E-82	2.35179E-81	80.62860212	down
gene-PFY09_RS17760	-1.087337805	3.91438E-82	3.9015E-81	80.40876806	down
gene-PFY09_RS16310	-2.101103795	1.82577E-81	1.79032E-80	79.74706974	down
gene-PFY09_RS20425	-1.588950919	1.52784E-80	1.47903E-79	78.83002329	down
gene-PFY09_RS08370	-3.239825042	2.99614E-80	2.89118E-79	78.53892446	down

gene-PFY09_RS07775	-1.435629773	2.91608E-78	2.75256E-77	76.56026363	down
gene-PFY09_RS13965	-1.824842606	3.58648E-76	3.3437E-75	74.47577286	down
gene-PFY09_RS08355	-1.625944708	2.57295E-74	2.36961E-73	72.62532241	down
gene-PFY09_RS00415	-2.596276319	5.35784E-74	4.91948E-73	72.30808119	down
gene-PFY09_RS09375	-1.237669799	6.21576E-72	5.5231E-71	70.2578172	down
gene-PFY09_RS07745	-2.100352416	6.58504E-72	5.83411E-71	70.23402511	down
gene-PFY09_RS07990	-1.15818832	2.36356E-71	2.07582E-70	69.68280975	down
gene-PFY09_RS16315	-1.939305225	9.01041E-71	7.84527E-70	69.10539202	down
gene-PFY09_RS01825	-1.934543997	5.71613E-70	4.93444E-69	68.30676243	down
gene-PFY09_RS01820	-1.868839807	1.00415E-69	8.64369E-69	68.06330057	down
gene-PFY09_RS07730	-1.569132926	1.07464E-68	9.1982E-68	67.03629697	down
gene-PFY09_RS20165	-2.785506802	3.71848E-67	3.13843E-66	65.5032869	down
gene-PFY09_RS02660	-2.706275679	1.42272E-66	1.19414E-65	64.92294522	down
gene-PFY09_RS06280	-3.086816894	3.39132E-66	2.83077E-65	64.5480949	down
gene-PFY09_RS06835	-1.795935729	3.60664E-63	2.95355E-62	61.52965613	down
gene-PFY09_RS09865	-1.042954781	1.60556E-62	1.30425E-61	60.88463923	down
gene-PFY09_RS12770	-2.581410075	1.99603E-62	1.6171E-61	60.79126182	down
gene-PFY09_RS06270	-2.084769005	3.19455E-62	2.57433E-61	60.58933541	down
gene-PFY09_RS00150	-1.850016477	7.58553E-62	6.09659E-61	60.21491282	down
gene-PFY09_RS10020	-2.532739065	6.09414E-59	4.73468E-58	57.32470977	down
gene-PFY09_RS00075	-1.059714009	1.09033E-58	8.44938E-58	57.07317495	down
gene-PFY09_RS11945	-1.947154343	3.76754E-58	2.89004E-57	56.53909602	down

gene-PFY09_RS04715	-2.365157085	5.08138E-57	3.87823E-56	55.41136599	down
gene-PFY09_RS15485	-3.733672942	2.17837E-56	1.646E-55	54.78357056	down
gene-PFY09_RS02380	-1.932552494	3.35361E-56	2.52145E-55	54.59835047	down
gene-PFY09_RS01195	-1.634057377	3.62434E-55	2.68502E-54	53.57105234	down
gene-PFY09_RS18545	-1.499378625	3.8078E-55	2.81406E-54	53.55066668	down
gene-PFY09_RS19245	-1.096485065	4.57087E-55	3.36977E-54	53.47239991	down
gene-PFY09_RS17850	-1.081284026	7.05544E-53	5.10214E-52	51.29224743	down
gene-PFY09_RS15025	-1.427306229	1.09224E-51	7.76874E-51	50.10964959	down
gene-PFY09_RS12835	-1.057223682	6.3511E-51	4.47531E-50	49.34917669	down
gene-PFY09_RS01515	-1.897147059	1.92217E-50	1.34198E-49	48.87225493	down
gene-PFY09_RS07600	-1.580875226	1.48311E-49	1.02132E-48	47.99083746	down
gene-PFY09_RS00670	-1.576245241	1.79695E-47	1.19403E-46	45.92298604	down
gene-PFY09_RS12100	-1.133599047	5.14696E-47	3.40508E-46	45.46787212	down
gene-PFY09_RS14500	-1.57694857	9.42071E-47	6.20538E-46	45.20723159	down
gene-PFY09_RS07175	-1.704141046	5.46816E-46	3.55548E-45	44.44910236	down
gene-PFY09_RS15140	-1.145100155	1.4012E-45	9.0719E-45	44.04230198	down
gene-PFY09_RS01375	-1.74691199	2.48014E-45	1.5989E-44	43.79617887	down
gene-PFY09_RS02905	-1.434461533	2.74087E-45	1.76323E-44	43.75369026	down
gene-PFY09_RS17855	-1.62900798	6.69287E-45	4.26038E-44	43.37055173	down
gene-PFY09_RS12780	-2.813166298	1.11748E-44	7.06884E-44	43.15065205	down
gene-PFY09_RS08665	-2.239466402	2.32185E-44	1.46262E-43	42.83486907	down
gene-PFY09_RS10015	-1.053619325	3.08151E-43	1.93713E-42	41.71284064	down

gene-PFY09_RS18785	-1.573292612	4.03773E-43	2.52254E-42	41.59816182	down
gene-PFY09_RS07030	-1.853527289	5.05494E-43	3.15154E-42	41.50147745	down
gene-PFY09_RS15135	-1.82188517	3.56557E-42	2.20034E-41	40.65750994	down
gene-PFY09_RS13970	-3.617564415	3.88465E-42	2.39238E-41	40.62117003	down
gene-PFY09_RS03600	-1.236550567	1.8282E-41	1.11682E-40	39.95201611	down
gene-PFY09_RS02855	-2.053485294	2.11767E-41	1.29106E-40	39.88905494	down
gene-PFY09_RS18790	-1.5979338	5.70453E-41	3.44317E-40	39.46304099	down
gene-PFY09_RS10600	-1.081653766	6.19553E-41	3.7321E-40	39.42804687	down
gene-PFY09_RS20740	-2.112749594	8.99827E-41	5.40967E-40	39.26682912	down
gene-PFY09_RS09090	-1.164021985	2.23646E-40	1.33658E-39	38.87400368	down
gene-PFY09_RS07795	-2.064503399	1.92113E-39	1.1325E-38	37.94596322	down
gene-PFY09_RS18795	-1.339805838	2.76574E-39	1.62093E-38	37.7902364	down
gene-PFY09_RS11055	-1.57601122	6.9439E-39	4.04616E-38	37.39295714	down
gene-PFY09_RS10705	-1.534599632	9.49651E-39	5.4913E-38	37.26032457	down
gene-PFY09_RS20175	-1.263743054	2.09823E-38	1.20638E-37	36.91851586	down
gene-PFY09_RS19000	-1.613288033	8.27463E-38	4.71281E-37	36.32672045	down
gene-PFY09_RS12840	-1.412200253	1.46075E-37	8.28855E-37	36.08152141	down
gene-PFY09_RS17870	-2.172044439	1.69316E-37	9.58929E-37	36.01821358	down
gene-PFY09_RS19490	-1.60898691	6.94511E-37	3.89698E-36	35.40927181	down
gene-PFY09_RS05445	-1.116441604	8.98279E-37	5.02175E-36	35.2991452	down
gene-PFY09_RS14795	-1.440723463	4.3562E-36	2.41303E-35	34.61743672	down
gene-PFY09_RS01575	-1.198457988	6.09578E-36	3.37048E-35	34.47230847	down

gene-PFY09_RS03075	-1.245207504	2.06482E-35	1.1334E-34	33.94561522	down
gene-PFY09_RS02315	-3.179475727	1.42413E-34	7.73318E-34	33.11164196	down
gene-PFY09_RS02000	-1.272116312	2.20146E-34	1.19328E-33	32.92325831	down
gene-PFY09_RS20090	-1.356967706	1.05306E-33	5.59784E-33	32.25197961	down
gene-PFY09_RS06820	-3.83651173	1.24892E-33	6.62738E-33	32.17865781	down
gene-PFY09_RS03920	-1.671046737	2.95888E-33	1.56464E-32	31.80558429	down
gene-PFY09_RS01870	-2.032116002	8.16934E-33	4.29741E-32	31.36679305	down
gene-PFY09_RS13015	-1.957806534	3.31145E-32	1.724E-31	30.7634622	down
gene-PFY09_RS11355	-1.294235332	6.84974E-32	3.53572E-31	30.45152167	down
gene-PFY09_RS15785	-2.196490187	1.00262E-31	5.14907E-31	30.28827151	down
gene-PFY09_RS20630	-1.027998934	1.99312E-31	1.01498E-30	29.99354183	down
gene-PFY09_RS02060	-1.290431721	2.92115E-31	1.4826E-30	29.82897711	down
gene-PFY09_RS13900	-1.227783872	4.27222E-31	2.16107E-30	29.66533042	down
gene-PFY09_RS13950	-1.794269595	7.21546E-31	3.6317E-30	29.43988984	down
gene-PFY09_RS20670	-1.631180077	1.16164E-30	5.81782E-30	29.23523979	down
gene-PFY09_RS10880	-2.641355227	2.85846E-30	1.42688E-29	28.84561388	down
gene-PFY09_RS03470	-1.393418416	3.2472E-30	1.61296E-29	28.79237754	down
gene-PFY09_RS18115	-1.256003912	7.78661E-30	3.83633E-29	28.41608396	down
gene-PFY09_RS19335	-1.505549864	8.78725E-30	4.31529E-29	28.36498971	down
gene-PFY09_RS18695	-1.980698756	6.84121E-29	3.28508E-28	27.48345353	down
gene-PFY09_RS12890	-1.447313637	1.03843E-28	4.97854E-28	27.30289775	down
gene-PFY09_RS07020	-1.517591797	1.13084E-28	5.41303E-28	27.26655952	down

gene-PFY09_RS11360	-1.290725826	2.30933E-28	1.09503E-27	26.9605729	down
gene-PFY09_RS02515	-2.077666267	3.49416E-28	1.65169E-27	26.78207235	down
gene-PFY09_RS02215	-1.419578243	4.06975E-28	1.92077E-27	26.71652452	down
gene-PFY09_RS07655	-1.916033572	1.10837E-27	5.15876E-27	26.2874545	down
gene-PFY09_RS07765	-2.006462702	1.11778E-27	5.1946E-27	26.28444773	down
gene-PFY09_RS08650	-1.631521407	2.00662E-27	9.31095E-27	26.03100577	down
gene-PFY09_RS11295	-1.087181684	3.07103E-27	1.42064E-26	25.84751456	down
gene-PFY09_RS19695	-2.322916447	3.67055E-27	1.69281E-26	25.77139171	down
gene-PFY09_RS11960	-1.047011726	3.85848E-27	1.77678E-26	25.75036719	down
gene-PFY09_RS16125	-1.654677304	9.33777E-27	4.2804E-26	25.36851577	down
gene-PFY09_RS02210	-2.329761776	1.02347E-26	4.67738E-26	25.32999742	down
gene-PFY09_RS02835	-1.129296136	1.02979E-26	4.69921E-26	25.32797476	down
gene-PFY09_RS19330	-1.405417758	1.01143E-25	4.5268E-25	24.3442083	down
gene-PFY09_RS02065	-1.017325471	4.91688E-25	2.15915E-24	23.66571679	down
gene-PFY09_RS18440	-1.556997225	6.01029E-25	2.63548E-24	23.57913998	down
gene-PFY09_RS19485	-1.532451309	1.38524E-24	6.02192E-24	23.22026513	down
gene-PFY09_RS11065	-1.967603607	4.08502E-24	1.76069E-23	22.75431817	down
gene-PFY09_RS02135	-1.602629082	4.70897E-24	2.02673E-23	22.69320416	down
gene-PFY09_RS20655	-1.598836568	7.10274E-24	3.04403E-23	22.51655101	down
gene-PFY09_RS17590	-1.132412114	1.22283E-23	5.2112E-23	22.28306263	down
gene-PFY09_RS07705	-1.206030853	1.42991E-23	6.07661E-23	22.21633837	down
gene-PFY09_RS10005	-1.089687041	1.57099E-23	6.65747E-23	22.17669081	down

gene-PFY09_RS07025	-1.647034667	5.72389E-23	2.41215E-22	21.61759495	down
gene-PFY09_RS19700	-1.986458079	1.07597E-22	4.50926E-22	21.34589425	down
gene-PFY09_RS16500	-1.218764821	1.84939E-22	7.67623E-22	21.11485197	down
gene-PFY09_RS06990	-1.763095611	1.86463E-22	7.7289E-22	21.11188226	down
gene-PFY09_RS02635	-1.976045233	3.45222E-22	1.4193E-21	20.84792633	down
gene-PFY09_RS12755	-4.778055494	5.56771E-22	2.27668E-21	20.64269883	down
gene-PFY09_RS02295	-2.004592168	9.08361E-22	3.68452E-21	20.43361944	down
gene-PFY09_RS01170	-1.436135806	1.21368E-21	4.90982E-21	20.30893456	down
gene-PFY09_RS01495	-1.20379721	5.31577E-21	2.10546E-20	19.67665284	down
gene-PFY09_RS03385	-2.268858972	6.78754E-21	2.68139E-20	19.5716402	down
gene-PFY09_RS13585	-1.788544747	6.84741E-21	2.70152E-20	19.56839226	down
gene-PFY09_RS14905	-1.412467994	1.53877E-20	6.03167E-20	19.21956259	down
gene-PFY09_RS02320	-1.676894302	2.88241E-20	1.12403E-19	18.94922266	down
gene-PFY09_RS11260	-1.972694	3.84738E-20	1.49265E-19	18.82604293	down
gene-PFY09_RS11245	-1.23639264	4.38231E-20	1.69367E-19	18.77117079	down
gene-PFY09_RS06740	-1.709680492	6.14523E-20	2.36896E-19	18.62544209	down
gene-PFY09_RS12740	-3.012094566	7.12584E-20	2.74001E-19	18.56224749	down
gene-PFY09_RS01205	-1.211959002	9.01142E-20	3.45628E-19	18.46139114	down
gene-PFY09_RS13120	-1.127534579	1.38235E-19	5.28186E-19	18.27721351	down
gene-PFY09_RS11250	-1.319441804	1.58764E-19	6.05863E-19	18.21762586	down
gene-PFY09_RS01230	-1.071317415	1.61253E-19	6.14587E-19	18.21141646	down
gene-PFY09_RS09920	-1.102731635	1.95538E-19	7.42455E-19	18.12932993	down

gene-PFY09_RS02200	-1.506623416	3.01564E-19	1.14075E-18	17.94280978	down
gene-PFY09_RS14665	-1.785316532	3.69218E-19	1.39319E-18	17.85599037	down
gene-PFY09_RS12750	-4.189961076	4.21009E-19	1.58664E-18	17.79952238	down
gene-PFY09_RS14365	-1.291985666	4.77618E-19	1.79551E-18	17.74581202	down
gene-PFY09_RS13020	-2.202063429	5.37291E-19	2.01509E-18	17.69570569	down
gene-PFY09_RS03115	-2.252447839	5.37357E-19	2.01509E-18	17.69570569	down
gene-PFY09_RS16460	-1.82075952	1.06459E-18	3.94824E-18	17.40359649	down
gene-PFY09_RS08640	-1.42055243	1.09623E-18	4.0606E-18	17.39140947	down
gene-PFY09_RS09105	-3.590410859	1.48121E-18	5.45993E-18	17.26281322	down
gene-PFY09_RS12880	-2.101640851	1.52581E-18	5.61751E-18	17.25045616	down
gene-PFY09_RS02350	-1.174624828	1.77568E-18	6.52949E-18	17.18512065	down
gene-PFY09_RS15650	-2.978712378	1.92647E-18	7.06682E-18	17.15077589	down
gene-PFY09_RS02205	-1.85293682	2.59945E-18	9.51249E-18	17.02170564	down
gene-PFY09_RS02375	-1.40318302	3.32305E-18	1.2102E-17	16.91714391	down
gene-PFY09_RS07685	-1.163128102	5.40776E-18	1.95765E-17	16.70826546	down
gene-PFY09_RS02450	-1.311307229	1.35219E-17	4.85444E-17	16.31386089	down
gene-PFY09_RS07760	-2.233104013	3.38745E-17	1.20328E-16	15.91963387	down
gene-PFY09_RS15100	-1.257496228	4.63668E-17	1.64125E-16	15.78482448	down
gene-PFY09_RS08325	-1.468770891	8.91923E-17	3.12792E-16	15.50474384	down
gene-PFY09_RS17625	-1.239143731	1.43092E-16	4.97781E-16	15.30296145	down
gene-PFY09_RS15860	-1.302182344	1.44095E-16	5.00699E-16	15.30042356	down
gene-PFY09_RS04650	-1.651706336	4.90802E-16	1.67658E-15	14.77557464	down

gene-PFY09_RS02670	-1.082799122	7.65672E-16	2.60673E-15	14.58390467	down
gene-PFY09_RS20500	-1.492083407	8.30631E-16	2.82154E-15	14.54951424	down
gene-PFY09_RS06455	-1.775276588	1.04295E-15	3.53087E-15	14.45211827	down
gene-PFY09_RS15285	-1.709959211	1.05304E-15	3.56108E-15	14.4484188	down
gene-PFY09_RS01865	-1.907725613	1.79889E-15	6.01615E-15	14.22068148	down
gene-PFY09_RS16420	-1.178196098	1.88168E-15	6.27919E-15	14.20209638	down
gene-PFY09_RS12765	-2.236203735	3.10288E-15	1.03089E-14	13.98678775	down
gene-PFY09_RS04905	-2.04273764	3.54052E-15	1.17372E-14	13.93043671	down
gene-PFY09_RS15925	-2.329293875	5.14119E-15	1.69693E-14	13.77033617	down
gene-PFY09_RS13855	-1.199085371	5.78334E-15	1.90473E-14	13.7201663	down
gene-PFY09_RS15495	-2.589072973	7.01425E-15	2.30762E-14	13.63683592	down
gene-PFY09_RS15300	-1.498133809	8.17855E-15	2.68774E-14	13.57061223	down
gene-PFY09_RS02330	-2.160226858	1.52714E-14	4.98625E-14	13.30222605	down
gene-PFY09_RS20640	-1.395417272	1.62525E-14	5.30087E-14	13.27565289	down
gene-PFY09_RS08155	-1.719152235	1.67298E-14	5.44483E-14	13.26401536	down
gene-PFY09_RS07790	-2.11227018	2.41714E-14	7.83309E-14	13.10606691	down
gene-PFY09_RS15750	-1.188543168	2.43025E-14	7.86716E-14	13.10418206	down
gene-PFY09_RS05385	-2.484509976	3.53763E-14	1.13911E-13	12.94343514	down
gene-PFY09_RS01715	-2.145672566	3.61198E-14	1.16182E-13	12.93486293	down
gene-PFY09_RS02890	-1.580420214	4.50173E-14	1.44188E-13	12.84106949	down
gene-PFY09_RS07755	-1.137354161	7.80624E-14	2.48194E-13	12.6052085	down
gene-PFY09_RS20645	-1.522257986	9.1646E-14	2.91077E-13	12.53599204	down

gene-PFY09_RS15500	-2.324700217	1.0855E-13	3.43326E-13	12.46429364	down
gene-PFY09_RS04960	-1.064388576	1.17393E-13	3.70522E-13	12.43118641	down
gene-PFY09_RS15970	-1.006577766	1.7111E-13	5.37266E-13	12.26981037	down
gene-PFY09_RS08625	-1.770754378	2.37591E-13	7.4064E-13	12.13039288	down
gene-PFY09_RS03590	-3.90618024	3.74495E-13	1.16381E-12	11.93411646	down
gene-PFY09_RS07495	-1.903380208	7.34906E-13	2.26528E-12	11.64487896	down
gene-PFY09_RS11490	-1.340400427	1.59701E-12	4.88783E-12	11.31088396	down
gene-PFY09_RS19450	-1.401073105	2.01308E-12	6.15502E-12	11.21077042	down
gene-PFY09_RS14690	-1.561543101	2.17767E-12	6.64485E-12	11.17751478	down
gene-PFY09_RS15395	-1.111496088	2.32257E-12	7.0515E-12	11.15171838	down
gene-PFY09_RS12735	-2.487078435	2.83472E-12	8.57206E-12	11.06691474	down
gene-PFY09_RS18780	-2.675607714	3.06412E-12	9.25651E-12	11.03355263	down
gene-PFY09_RS10885	-1.04933257	3.40814E-12	1.02651E-11	10.98863836	down
gene-PFY09_RS18775	-1.122407707	3.48738E-12	1.04933E-11	10.97908818	down
gene-PFY09_RS03155	-1.867939233	4.08857E-12	1.22536E-11	10.91173758	down
gene-PFY09_RS10210	-1.491920146	4.22606E-12	1.26531E-11	10.89780199	down
gene-PFY09_RS01860	-1.298624463	4.66319E-12	1.39482E-11	10.85548325	down
gene-PFY09_RS15295	-3.272462703	8.04482E-12	2.37349E-11	10.62461174	down
gene-PFY09_RS18965	-1.239830468	8.24533E-12	2.43029E-11	10.61434253	down
gene-PFY09_RS14675	-1.744239514	8.86547E-12	2.61053E-11	10.58327102	down
gene-PFY09_RS20130	-1.58915688	1.08885E-11	3.19692E-11	10.49526814	down
gene-PFY09_RS10480	-1.325263556	2.46065E-11	7.1075E-11	10.14828333	down

gene-PFY09_RS20110	-1.803993889	2.76281E-11	7.94244E-11	10.10004621	down
gene-PFY09_RS17940	-1.59773618	3.54546E-11	1.01347E-10	9.994190196	down
gene-PFY09_RS18960	-1.031546509	3.56324E-11	1.01759E-10	9.992427302	down
gene-PFY09_RS11500	-1.126699046	3.93649E-11	1.12312E-10	9.949572232	down
gene-PFY09_RS03395	-1.549547574	5.5343E-11	1.56749E-10	9.804794877	down
gene-PFY09_RS02840	-1.170195425	6.53661E-11	1.84757E-10	9.733399601	down
gene-PFY09_RS14565	-1.17308674	6.76874E-11	1.9114E-10	9.718649516	down
gene-PFY09_RS07720	-1.420318402	7.52316E-11	2.12246E-10	9.673161399	down
gene-PFY09_RS06360	-1.59413883	1.03829E-10	2.91838E-10	9.534858841	down
gene-PFY09_RS19640	-1.050405793	1.40426E-10	3.92519E-10	9.40613898	down
gene-PFY09_RS14645	-1.612078306	1.46518E-10	4.08793E-10	9.388496745	down
gene-PFY09_RS09200	-1.696511589	1.66338E-10	4.62389E-10	9.334992079	down
gene-PFY09_RS04970	-1.834808923	2.21697E-10	6.12903E-10	9.212608454	down
gene-PFY09_RS04260	-2.565079393	2.2854E-10	6.30671E-10	9.200197086	down
gene-PFY09_RS05680	-1.717733552	2.86773E-10	7.88497E-10	9.103200033	down
gene-PFY09_RS01660	-1.173521424	3.13156E-10	8.57149E-10	9.066943612	down
gene-PFY09_RS14585	-1.827655677	3.2957E-10	9.00449E-10	9.04554077	down
gene-PFY09_RS20445	-1.759223811	3.73133E-10	1.01855E-09	8.992017062	down
gene-PFY09_RS08320	-2.009034217	4.37351E-10	1.19277E-09	8.923441581	down
gene-PFY09_RS19135	-1.542379567	8.0174E-10	2.1517E-09	8.667217492	down
gene-PFY09_RS13905	-2.201425742	8.42807E-10	2.25592E-09	8.646675918	down
gene-PFY09_RS14640	-1.95646111	1.09433E-09	2.89593E-09	8.53821258	down

gene-PFY09_RS02920	-1.052630588	1.7415E-09	4.56465E-09	8.340592707	down
gene-PFY09_RS04770	-1.030005565	1.82115E-09	4.76932E-09	8.32154383	down
gene-PFY09_RS10970	-1.215699417	1.89665E-09	4.95418E-09	8.305028599	down
gene-PFY09_RS03925	-1.331643602	2.17025E-09	5.64296E-09	8.248493267	down
gene-PFY09_RS08770	-1.286379	2.17151E-09	5.64296E-09	8.248493267	down
gene-PFY09_RS02915	-1.322078314	2.2357E-09	5.79979E-09	8.236587532	down
gene-PFY09_RS02180	-1.756374936	2.50029E-09	6.46405E-09	8.189495394	down
gene-PFY09_RS04210	-1.042650304	3.02836E-09	7.80266E-09	8.107757541	down
gene-PFY09_RS13595	-1.80955441	3.17511E-09	8.17382E-09	8.087575175	down
gene-PFY09_RS14600	-1.278436998	3.27396E-09	8.40784E-09	8.075315483	down
gene-PFY09_RS07780	-1.322160265	3.27434E-09	8.40784E-09	8.075315483	down
gene-PFY09_RS12255	-1.078008876	3.86009E-09	9.87844E-09	8.005311511	down
gene-PFY09_RS15865	-1.461248285	3.87579E-09	9.91025E-09	8.00391541	down
gene-PFY09_RS01940	-2.045194434	4.20814E-09	1.0751E-08	7.968551515	down
gene-PFY09_RS14635	-1.239220532	4.48464E-09	1.14189E-08	7.94237682	down
gene-PFY09_RS12830	-1.334156393	5.80114E-09	1.47092E-08	7.832411605	down
gene-PFY09_RS01630	-1.264332683	8.54193E-09	2.15146E-08	7.66726692	down
gene-PFY09_RS10965	-1.268392405	9.93603E-09	2.49637E-08	7.602691765	down
gene-PFY09_RS14095	-1.92862526	1.01039E-08	2.53643E-08	7.595776863	down
gene-PFY09_RS02555	-1.261265326	1.38603E-08	3.45938E-08	7.461001892	down
gene-PFY09_RS04965	-1.153136993	1.98266E-08	4.92011E-08	7.308025535	down
gene-PFY09_RS07695	-1.231692234	2.68193E-08	6.61746E-08	7.1793089	down

gene-PFY09_RS10750	-3.697988004	4.56203E-08	1.11655E-07	6.952120171	down
gene-PFY09_RS01600	-1.698853697	4.73265E-08	1.15644E-07	6.936875078	down
gene-PFY09_RS11310	-2.017835509	5.27011E-08	1.28467E-07	6.891209112	down
gene-PFY09_RS11300	-2.904488843	6.2495E-08	1.51852E-07	6.818578675	down
gene-PFY09_RS06320	-1.091653621	7.86298E-08	1.90142E-07	6.720921015	down
gene-PFY09_RS15375	-1.19259787	8.31355E-08	2.00718E-07	6.697414552	down
gene-PFY09_RS01165	-2.215350258	9.20199E-08	2.21814E-07	6.654010747	down
gene-PFY09_RS15130	-2.469252066	9.30956E-08	2.2405E-07	6.649654135	down
gene-PFY09_RS10640	-1.084519264	9.31988E-08	2.24121E-07	6.649517695	down
gene-PFY09_RS06525	-1.140926442	1.07501E-07	2.57697E-07	6.5888904	down
gene-PFY09_RS14655	-1.597180902	1.37108E-07	3.27117E-07	6.485297522	down
gene-PFY09_RS14670	-1.137954487	1.40675E-07	3.35361E-07	6.474487092	down
gene-PFY09_RS11325	-1.044722809	1.82374E-07	4.34087E-07	6.362423188	down
gene-PFY09_RS03405	-1.106636359	2.01151E-07	4.7728E-07	6.321226674	down
gene-PFY09_RS18670	-1.455834147	2.28422E-07	5.39979E-07	6.26762291	down
gene-PFY09_RS02810	-1.035390693	2.31291E-07	5.45803E-07	6.262964138	down
gene-PFY09_RS07215	-1.316411551	2.33535E-07	5.50671E-07	6.259107708	down
gene-PFY09_RS08845	-1.752660301	3.14321E-07	7.36007E-07	6.13311779	down
gene-PFY09_RS01950	-1.239309258	3.30917E-07	7.73672E-07	6.111442877	down
gene-PFY09_RS02640	-1.247711266	3.503E-07	8.17097E-07	6.087726124	down
gene-PFY09_RS20515	-1.416749134	3.91834E-07	9.12573E-07	6.039732263	down
gene-PFY09_RS12995	-1.085385795	4.64121E-07	1.07432E-06	5.968866214	down

gene-PFY09_RS20045	-1.00754223	4.67857E-07	1.08132E-06	5.966047037	down
gene-PFY09_RS12230	-1.189382408	4.89542E-07	1.13057E-06	5.946701618	down
gene-PFY09_RS07750	-2.513464036	5.18873E-07	1.19649E-06	5.922092013	down
gene-PFY09_RS20180	-1.41335428	5.83263E-07	1.34089E-06	5.872608176	down
gene-PFY09_RS08015	-1.872536367	6.05159E-07	1.38911E-06	5.857262052	down
gene-PFY09_RS16130	-1.141891675	6.76891E-07	1.54908E-06	5.809926311	down
gene-PFY09_RS05160	-1.486091953	7.23852E-07	1.6553E-06	5.78112367	down
gene-PFY09_RS12410	-1.647938256	7.43284E-07	1.69845E-06	5.76994623	down
gene-PFY09_RS06910	-1.59676349	1.01892E-06	2.3126E-06	5.635898687	down
gene-PFY09_RS06515	-2.497154718	1.05909E-06	2.40198E-06	5.619431097	down
gene-PFY09_RS14950	-1.052185847	1.29287E-06	2.9256E-06	5.533784768	down
gene-PFY09_RS19005	-1.015704973	1.34134E-06	3.03077E-06	5.518447597	down
gene-PFY09_RS18080	-1.268205971	1.35077E-06	3.04754E-06	5.516050652	down
gene-PFY09_RS06460	-1.31279661	1.60419E-06	3.60854E-06	5.442668308	down
gene-PFY09_RS04915	-2.283143737	1.77613E-06	3.98939E-06	5.399093838	down
gene-PFY09_RS04265	-1.950594944	1.77778E-06	3.99013E-06	5.399013472	down
gene-PFY09_RS01675	-3.764313159	1.87778E-06	4.20834E-06	5.375888749	down
gene-PFY09_RS11060	-1.559133806	2.13533E-06	4.77816E-06	5.320739739	down
gene-PFY09_RS02435	-1.60369783	2.19593E-06	4.90322E-06	5.309518786	down
gene-PFY09_RS11630	-2.490725125	2.51686E-06	5.6033E-06	5.251555892	down
gene-PFY09_RS20810	-1.159507445	2.66333E-06	5.92502E-06	5.227310027	down
gene-PFY09_RS15465	-2.708085744	2.73342E-06	6.0676E-06	5.216982913	down

gene-PFY09_RS19240	-1.271276885	2.86298E-06	6.35053E-06	5.197189926	down
gene-PFY09_RS02745	-1.562388704	3.25402E-06	7.19159E-06	5.143174917	down
gene-PFY09_RS06785	-1.593672363	3.39879E-06	7.49515E-06	5.125219439	down
gene-PFY09_RS10250	-2.07931206	4.32435E-06	9.4879E-06	5.022829969	down
gene-PFY09_RS17665	-1.393024477	4.6554E-06	1.01847E-05	4.992049786	down
gene-PFY09_RS01510	-1.518969583	4.94585E-06	1.07968E-05	4.966706238	down
gene-PFY09_RS11665	-1.128610454	6.00516E-06	1.30622E-05	4.883984157	down
gene-PFY09_RS19645	-1.285726423	6.07007E-06	1.31845E-05	4.879937703	down
gene-PFY09_RS18700	-1.405380043	7.67151E-06	1.65678E-05	4.780734437	down
gene-PFY09_RS11320	-1.014010665	8.19562E-06	1.76871E-05	4.75234261	down
gene-PFY09_RS10875	-1.084027215	9.33666E-06	2.0121E-05	4.696351005	down
gene-PFY09_RS03135	-1.171258637	9.55554E-06	2.0578E-05	4.686596452	down
gene-PFY09_RS07550	-2.591549418	9.59064E-06	2.0639E-05	4.68531225	down
gene-PFY09_RS01530	-1.391289419	1.03198E-05	2.21294E-05	4.655030049	down
gene-PFY09_RS17375	-1.2669786	1.22212E-05	2.60409E-05	4.584344418	down
gene-PFY09_RS20100	-1.143368036	1.53484E-05	3.2476E-05	4.488437925	down
gene-PFY09_RS11335	-1.024141624	1.55349E-05	3.28476E-05	4.483496336	down
gene-PFY09_RS11940	-1.883180542	1.63955E-05	3.46431E-05	4.460382701	down
gene-PFY09_RS15845	-1.057901338	1.66771E-05	3.51892E-05	4.453590649	down
gene-PFY09_RS11410	-1.911650943	1.70281E-05	3.59048E-05	4.444846977	down
gene-PFY09_RS14680	-1.617737614	1.75933E-05	3.7045E-05	4.431270936	down
gene-PFY09_RS11100	-1.257964031	2.08446E-05	4.36786E-05	4.359731597	down

gene-PFY09_RS20255	-1.051768378	2.09545E-05	4.38785E-05	4.357748415	down
gene-PFY09_RS04270	-2.551988797	3.42399E-05	7.10594E-05	4.148378335	down
gene-PFY09_RS11265	-1.571748344	4.2801E-05	8.82824E-05	4.054125753	down
gene-PFY09_RS08695	-1.098679138	4.68066E-05	9.62824E-05	4.016452984	down
gene-PFY09_RS01670	-1.36280183	4.78834E-05	9.83638E-05	4.00716485	down
gene-PFY09_RS03545	-1.071536432	4.80875E-05	9.87162E-05	4.005611572	down
gene-PFY09_RS04815	-1.289783004	5.24756E-05	0.000107433	3.968862024	down
gene-PFY09_RS08375	-1.406043347	5.78151E-05	0.000117966	3.928242647	down
gene-PFY09_RS01190	-1.355316548	6.24398E-05	0.000127231	3.895406922	down
gene-PFY09_RS14810	-1.252534764	6.47823E-05	0.000131827	3.879995536	down
gene-PFY09_RS09255	-1.158772839	6.75839E-05	0.00013716	3.862773622	down
gene-PFY09_RS15795	-1.132090766	6.8716E-05	0.000139271	3.856140603	down
gene-PFY09_RS18750	-1.509981533	8.61856E-05	0.000173632	3.760370263	down
gene-PFY09_RS13955	-2.138809443	8.71315E-05	0.000175421	3.755918719	down
gene-PFY09_RS04920	-1.442461899	8.99148E-05	0.000180664	3.743127354	down
gene-PFY09_RS08835	-1.819519924	9.38725E-05	0.000188242	3.725283679	down
gene-PFY09_RS17380	-1.098910067	9.789E-05	0.00019578	3.708231559	down
gene-PFY09_RS00585	-1.535488938	0.000104485	0.000208557	3.680776145	down
gene-PFY09_RS16365	-1.864518704	0.000127573	0.000253472	3.596070076	down
gene-PFY09_RS02800	-1.54271656	0.000132699	0.000263485	3.579244124	down
gene-PFY09_RS16035	-1.411278544	0.000142326	0.000282046	3.549679899	down
gene-PFY09_RS11315	-1.533212136	0.000144555	0.000286275	3.543216218	down

gene-PFY09_RS14725	-1.023229029	0.000162541	0.000320638	3.493985112	down
gene-PFY09_RS20605	-1.16751952	0.000163159	0.000321648	3.492619148	down
gene-PFY09_RS20215	-1.207444867	0.00016707	0.00032893	3.482896957	down
gene-PFY09_RS04955	-1.392672154	0.000168356	0.000331245	3.479850052	down
gene-PFY09_RS11240	-1.496406733	0.000180236	0.000353243	3.45192642	down
gene-PFY09_RS00410	-1.724288214	0.000183756	0.00035991	3.443805706	down
gene-PFY09_RS08145	-1.530823905	0.000186262	0.000364582	3.438204342	down
gene-PFY09_RS14590	-1.782359054	0.000192535	0.000376618	3.424099049	down
gene-PFY09_RS18840	-1.252536153	0.000207354	0.000404561	3.39301647	down
gene-PFY09_RS08130	-1.555000904	0.0002111	0.00041081	3.386358775	down
gene-PFY09_RS18285	-4.562522734	0.000297692	0.000572702	3.242071479	down
gene-PFY09_RS11530	-1.214925317	0.000312238	0.000599925	3.221903333	down
gene-PFY09_RS11605	-1.116842186	0.000357862	0.000684547	3.164596669	down
gene-PFY09_RS16325	-1.356926486	0.000366221	0.000699213	3.155390554	down
gene-PFY09_RS19650	-1.433493978	0.000366802	0.000699881	3.154975762	down
gene-PFY09_RS18850	-1.93776237	0.000375171	0.000714949	3.145725074	down
gene-PFY09_RS15610	-1.334955832	0.000401422	0.000762575	3.117717381	down
gene-PFY09_RS20510	-1.120521417	0.000402532	0.000764205	3.116790271	down
gene-PFY09_RS00500	-1.958303586	0.000439058	0.000832506	3.079612701	down
gene-PFY09_RS12960	-1.193465262	0.000455889	0.00086388	3.063546568	down
gene-PFY09_RS08940	-1.115644836	0.000460249	0.000871596	3.059684666	down
gene-PFY09_RS19255	-1.130472698	0.000510552	0.000961449	3.017073731	down

gene-PFY09_RS04630	-1.27866086	0.000543007	0.001021298	2.990847552	down
gene-PFY09_RS03485	-1.407794779	0.000594868	0.001114688	2.952846867	down
gene-PFY09_RS04855	-2.872152428	0.000604737	0.001131082	2.946505768	down
gene-PFY09_RS07805	-1.073487119	0.00066047	0.001230766	2.909824621	down
gene-PFY09_RS11280	-1.000708212	0.000756185	0.001404807	2.852383398	down
gene-PFY09_RS14475	-2.351611491	0.000784273	0.001454313	2.837342166	down
gene-PFY09_RS14415	-1.33043507	0.000888371	0.001640319	2.785071679	down
gene-PFY09_RS01025	-1.935727208	0.001308196	0.002392175	2.62120707	down
gene-PFY09_RS06275	-2.397463703	0.001615636	0.002929609	2.533190326	down
gene-PFY09_RS18220	-1.376706136	0.001681726	0.003045804	2.516297989	down
gene-PFY09_RS06555	-4.260019033	0.001700931	0.003076908	2.511885544	down
gene-PFY09_RS11545	-2.688040429	0.001726491	0.003119661	2.50589254	down
gene-PFY09_RS19960	-1.371887833	0.001834341	0.003306396	2.48064509	down
gene-PFY09_RS16140	-1.217522777	0.002213741	0.003966668	2.401574137	down
gene-PFY09_RS05910	-1.281048977	0.002310341	0.004125122	2.384563222	down
gene-PFY09_RS02385	-1.443234733	0.00234293	0.004178385	2.378991498	down
gene-PFY09_RS00100	-1.415779707	0.002416341	0.004299186	2.366613791	down
gene-PFY09_RS11185	-1.113979496	0.002700248	0.004781853	2.320403741	down
gene-PFY09_RS10605	-1.744574124	0.002712569	0.004799795	2.318777321	down
gene-PFY09_RS10080	-1.344302976	0.002869928	0.005061631	2.295709545	down
gene-PFY09_RS14685	-2.898003392	0.003264945	0.005728306	2.241973779	down
gene-PFY09_RS00050	-1.016817216	0.003409191	0.005957236	2.224955159	down

gene-PFY09_RS12930	-1.369819247	0.003529404	0.006156645	2.210655872	down
gene-PFY09_RS02535	-1.148645532	0.003619447	0.006306455	2.200214698	down
gene-PFY09_RS20460	-1.568693617	0.00362914	0.006319709	2.19930293	down
gene-PFY09_RS08480	-2.148820134	0.003722159	0.006474248	2.188810646	down
gene-PFY09_RS14250	-1.270131458	0.003763032	0.006541587	2.184316859	down
gene-PFY09_RS01525	-1.211710078	0.003854764	0.006693373	2.174354982	down
gene-PFY09_RS06255	-1.173653986	0.00397082	0.006879123	2.16246695	down
gene-PFY09_RS03505	-1.258477485	0.004035819	0.006987732	2.155663747	down
gene-PFY09_RS18860	-2.215538267	0.004596033	0.00792149	2.101193125	down
gene-PFY09_RS07430	-2.11549168	0.004732686	0.008147749	2.088962367	down
gene-PFY09_RS01625	-2.063293512	0.004752839	0.008163889	2.088102883	down
gene-PFY09_RS15585	-1.158944063	0.005216364	0.008899539	2.050632473	down
gene-PFY09_RS04615	-2.091625968	0.006434363	0.010928319	1.961446646	down
gene-PFY09_RS09150	-1.140776081	0.006473023	0.010981668	1.959331697	down
gene-PFY09_RS15015	-1.557483459	0.006512614	0.011036476	1.957169566	down
gene-PFY09_RS09270	-1.443765307	0.006532076	0.01106327	1.956116482	down
gene-PFY09_RS07690	-3.978872931	0.006686977	0.011312977	1.94642311	down
gene-PFY09_RS08840	-1.871893817	0.006715219	0.011348084	1.945077465	down
gene-PFY09_RS15600	-1.027990589	0.007080715	0.01194575	1.922786568	down
gene-PFY09_RS09285	-3.783605705	0.007547519	0.012704991	1.896025643	down
gene-PFY09_RS19465	-1.590942026	0.007722714	0.012949542	1.88774559	down
gene-PFY09_RS02445	-1.25736052	0.007825391	0.013099964	1.882729911	down

gene-PFY09_RS11790	-1.549822302	0.008003472	0.013383289	1.873437141	down
gene-PFY09_RS09815	-1.121761769	0.008762014	0.014603356	1.835547322	down
gene-PFY09_RS13910	-2.220915023	0.009423478	0.015654133	1.805370977	down
gene-PFY09_RS15690	-2.051079475	0.00969888	0.016098955	1.793202308	down
gene-PFY09_RS12570	-1.872627224	0.009701879	0.016098955	1.793202308	down
gene-PFY09_RS19115	-Inf	0.009888037	0.016389909	1.78542347	down
gene-PFY09_RS01060	-1.074726509	0.010066137	0.016666882	1.778145638	down
gene-PFY09_RS08100	-1.283315578	0.010359797	0.017125032	1.76636861	down
gene-PFY09_RS07015	-Inf	0.010656368	0.017596073	1.754584244	down
gene-PFY09_RS12040	-1.099999759	0.012852342	0.021027319	1.677216091	down
gene-PFY09_RS02760	-1.625748397	0.013058461	0.021345967	1.670684168	down
gene-PFY09_RS11395	-Inf	0.013061196	0.021345967	1.670684168	down
gene-PFY09_RS09085	-Inf	0.013732879	0.022431604	1.649139679	down
gene-PFY09_RS03490	-1.657423479	0.014911244	0.024225774	1.61572233	down
gene-PFY09_RS06480	-1.416899156	0.016386281	0.026452015	1.577541242	down
gene-PFY09_RS09295	-1.611860799	0.016675696	0.026875644	1.570641124	down
gene-PFY09_RS04900	-2.005456101	0.016938737	0.027256704	1.564526666	down
gene-PFY09_RS14125	-1.02287713	0.017723269	0.028428536	1.546245505	down
gene-PFY09_RS20050	-1.733200914	0.018642055	0.029807613	1.525672804	down
gene-PFY09_RS06985	-1.772068741	0.018901709	0.030175014	1.520352517	down
gene-PFY09_RS14605	-1.177949066	0.019360805	0.030826715	1.511072752	down
gene-PFY09_RS01845	-1.303913892	0.019737536	0.031393561	1.503159413	down

gene-PFY09_RS04120	-2.258776048	0.021981753	0.034780528	1.458663831	down
gene-PFY09_RS00140	-1.161062637	0.022479668	0.035494212	1.449842459	down
gene-PFY09_RS05510	-1.365276515	0.024377679	0.038351177	1.416221297	down
gene-PFY09_RS06950	-1.096292492	0.024546562	0.038596826	1.413448408	down
gene-PFY09_RS19965	-1.482477644	0.025200654	0.039563721	1.402702866	down
gene-PFY09_RS12515	-1.292671734	0.025983423	0.040729318	1.390092866	down
gene-PFY09_RS19230	-1.963955339	0.027193155	0.042493688	1.371675579	down
gene-PFY09_RS00145	-1.673783906	0.027598493	0.043038309	1.366144801	down
gene-PFY09_RS06210	-1.74635093	0.030502431	0.047299062	1.325147471	down
gene-PFY09_RS01125	-1.122969752	0.030704591	0.047577121	1.322601845	down
gene-PFY09_RS09435	-2.463790722	0.030713151	0.047577121	1.322601845	down
gene-PFY09_RS09885	-1.605875161	0.032415816	0.050010144	1.300941895	down
gene-PFY09_RS11200	-1.983766434	0.033454122	0.051454817	1.288573961	down
gene-PFY09_RS18855	-2.33264542	0.036770462	0.056156502	1.250599956	down
gene-PFY09_RS12925	-2.204806746	0.038142589	0.058076404	1.236000282	down
gene-PFY09_RS08505	-1.536357527	0.038945241	0.059268749	1.227174237	down
gene-PFY09_RS11305	-1.677358773	0.039614958	0.060227458	1.220205466	down
gene-PFY09_RS05745	-1.161520921	0.041746709	0.063214657	1.199182214	down
gene-PFY09_RS20685	-1.230098002	0.043878863	0.066244621	1.178849382	down
gene-PFY09_RS03880	-1.733024964	0.047283394	0.071065815	1.148339257	down

Supplementary Table S1. 1,053 differentially expressed genes (DEGs) with $\log_2$(fold change) and $-\log_{10}$(padj).
